# Prognostic factors for colchicine prophylaxis-related adverse events when initiating allopurinol for gout: retrospective cohort study

**DOI:** 10.1093/rheumatology/keae229

**Published:** 2024-04-18

**Authors:** Ram Bajpai, Richard Partington, Sara Muller, Harry Forrester, Christian D Mallen, Lorna Clarson, Nishita Padmanabhan, Rebecca Whittle, Edward Roddy

**Affiliations:** School of Medicine, Keele University, Keele, UK; School of Medicine, Keele University, Keele, UK; School of Medicine, Keele University, Keele, UK; School of Medicine, Keele University, Keele, UK; School of Medicine, Keele University, Keele, UK; School of Medicine, Keele University, Keele, UK; School of Medicine, Keele University, Keele, UK; School of Medicine, Keele University, Keele, UK; Institute of Applied Health Research, College of Medical and Dental Sciences, University of Birmingham, Birmingham, UK; National Institute for Health and Care Research (NIHR) Birmingham Biomedical Research Centre, Birmingham, UK; School of Medicine, Keele University, Keele, UK; Haywood Academic Rheumatology Centre, Midlands Partnership University NHS Foundation Trust, Stoke-on-Trent, UK

**Keywords:** gout, colchicine prophylaxis, adverse events, urate-lowering therapy, prognostic factors

## Abstract

**Objectives:**

Colchicine is commonly used to prevent flares when starting urate-lowering therapy for gout. Patients with gout are frequently concurrently prescribed other medications (such as statins) that may interact with colchicine, increasing the risk of adverse events. The aim of this study was to describe potential prognostic factors for adverse events in patients prescribed colchicine when initiating allopurinol.

**Methods:**

We conducted a retrospective cohort study in linked UK Clinical Practice Research Datalink and Hospital Episode Statistics datasets. Adults initiating allopurinol for gout with colchicine (1 April 1997 to 30 November 2016) were included. Potential prognostic factors were defined, and the likelihood of adverse events, including diarrhoea, nausea or vomiting, myocardial infarction, neuropathy, myalgia, myopathy, rhabdomyolysis and bone marrow suppression, were estimated.

**Results:**

From 1 April 1997 to 30 November 2016, 13 945 people with gout initiated allopurinol with colchicine prophylaxis [mean age 63.9 (s.d. 14.7) years, 78.2% male]. One-quarter (26%, 95% CI 25%, 27%) were prescribed one or more potentially interacting medicines, most commonly statins (21%, 95% CI 20%, 22%). Statins were not associated with increased adverse events, although other drugs were associated with some adverse outcomes. Diarrhoea and myocardial infarction were associated with more comorbidities and more severe chronic kidney disease.

**Conclusion:**

People were given colchicine prophylaxis despite commonly having preexisting prescriptions for medications with potential to interact with colchicine. Adverse events were more common in people who had more comorbidities and certain potentially interacting medications. Our findings will provide much-needed information about prognostic factors for colchicine-related adverse events that can inform treatment decisions about prophylaxis when initiating allopurinol.

Rheumatology key messages

- Colchicine prophylaxis was commonly prescribed in people already taking medications with potential to interact with colchicine.
- Gastrointestinal adverse events were more common in females, older people, and those taking amiodarone and with more comorbidities.
- Overall statin use was not associated with adverse events.

## Introduction

Gout is caused by high serum urate levels leading to the formation of MSU crystals in and around joints. It is the most prevalent inflammatory arthritis and affects 2.5% of adults in the UK and 3.9% in the USA [1, 2]. Gout is characterized by recurrent flares of excruciating joint pain and inflammation, and is associated with substantial comorbidity and impaired health-related quality of life [3, 4]. Urate-lowering therapy (ULT) for gout leads to gradual dissolution of MSU crystals and cessation of flares over several months [5–8]. However, initiating or escalating the dose of ULT often precipitates a gout flare, which may lead patients or practitioners to stop ULT, as they believe that it has exacerbated the gout [9]. Anti-inflammatory prophylaxis to prevent such flares, most commonly with colchicine, is therefore recommended for several months when initiating ULT [5–8].

Diarrhoea, nausea and vomiting are common side-effects of colchicine, but rarely, it can cause more serious side-effects such as myopathy, rhabdomyolysis, neuropathy or bone marrow suppression. We recently reported a cohort study which determined the risk of adverse events severe enough to warrant seeking healthcare associated with colchicine prophylaxis when initiating allopurinol in patients with gout in UK primary care [10]. We found that adverse events affected 1 in 15 people taking colchicine. However, serious adverse events were rare.

The risk of side-effects due to colchicine prophylaxis is thought to be influenced by patient characteristics. For example, the risk of serious events including neuromyopathy, rhabdomyolysis and bone marrow suppression is higher in people with chronic kidney disease (CKD) [11], which affects one in four people with gout [12], and in people taking certain medications. Through its metabolism by CYP3A4, colchicine toxicity is theoretically increased by concomitant treatment with common drugs such as statins, fibrates, macrolide antibiotics, rate-limiting calcium-channel blockers (verapamil, diltiazem), digoxin, amiodarone and antifungals, as well as protease inhibitors and ciclosporin [13]. Although colchicine is contraindicated in patients taking these medications, in our clinical experience from primary and secondary care, their co-prescription still commonly occurs. However, evidence that these factors are prognostic for adverse events is largely anecdotal or theoretical, and large research studies are lacking [14, 15].

In this study, we aimed to assess to what degree co-prescriptions and other potential prognostic factors for adverse events, including age, gender, BMI, CKD and multimorbidity, affect the risk of adverse events when commencing colchicine prophylaxis for ULT in people with gout.

## Methods

### Data source

We used data from the Clinical Practice Research Datalink (CPRD, December 2019) GOLD and Aurum datasets, which have >3 and 13 million patients, respectively, currently registered and research acceptable. CPRD contains electronic, coded information collected during the course of routine primary healthcare, is representative of the UK population in terms of age, sex and ethnicity, and has been used extensively for primary care research [16, 17]. We used the primary care data linked to the Hospital Episode Statistics (HES) Admitted Patient Care dataset, which has coverage from April 1997 to the present. The study was approved by CPRD’s in-house Independent Scientific Advisory Committee (ISAC) (protocol number: 19_233).

### Study design and participants

Retrospective cohort studies were assembled in GOLD and Aurum separately. Each consisted of people aged 18 years and over with a clinical code for gout between 1 April 1997 and 30 November 2016 (to allow HES linkage at the time of data access) who received a prescription for colchicine and on the same date received a prescription for allopurinol and did not receive a prescription for NSAID or glucocorticoids in the preceding month. The positive predictive value of a diagnosis of gout in CPRD has been shown to be 90% [18]. The definition of ULT was restricted to allopurinol since this is by far the most commonly prescribed ULT in UK primary care, accounting for 99% of first ULT prescriptions [19, 20].

The index date (i.e. start of time at risk) was the date of the concomitant first time prescriptions for allopurinol and colchicine following the diagnostic code for gout. Everyone was followed up for 6 months or until the end of colchicine treatment, whichever was sooner. The end of treatment was defined as 56 days after the last colchicine prescription.

### Adverse outcomes

We defined incident adverse events using clinical codes [Read/ Systematized Nomenclature of Medicine (SNOMED) in primary care; International Classification of Diseases (ICD) in secondary care] during the period of colchicine treatment. Events of interest were diarrhoea, nausea or vomiting, myocardial infarction (MI), neuropathy, myalgia, myopathy, rhabdomyolysis and bone marrow suppression. Each adverse event was analysed separately. Patients were excluded from the analysis if they had a Read/SNOMED/ICD code for the outcome in question in the 3 months preceding the index date. Consistent with the accepted practice for large database research, we assumed that the absence of a recorded adverse event meant that the patient did not consult for that condition or that if they did, the clinician did not think it was of sufficient relevance to record in the coded data. Code lists for all outcomes, exposures and covariates were compiled by a general practitioner and a rheumatologist and are available at https://researchdata.keele.ac.uk/67/.

### Potentially interacting prescriptions

Prescriptions for medications that could potentially interact with colchicine (statins, fibrates, verapamil, diltiazem, digoxin, amiodarone, oral ketoconazole and macrolide antibiotics) in the 30 days before the index date were considered potentially interacting prescriptions.

### Potential prognostic factors

We considered the following patient factors that might be associated with the rate of adverse outcomes: age in quartiles, sex, Charlson comorbidity index (scored 0, 1, 2, 3, 4+) [21], BMI [categorized as normal (18.5–24.9 kg/m^2^), overweight (25–29.9 kg/m^2^) and obese (≥30 kg/m^2^)], CKD category [1–2 (estimated glomerular filtration rate, eGFR ≥60 mL/min), 3 (eGFR 30–59 mL/min), 4–5 (eGFR <30 mL/min) defined by diagnostic codes or a record for eGFR <60 mL/min] and potentially interacting prescriptions (defined above). All were defined before the index date.

### Statistical analysis

The proportion (95% CI) of people receiving one or more and two or more potentially interacting prescription in the period 30 days before the index date was calculated, as was the proportion receiving colchicine in the presence of each named potentially interacting drug separately. These analyses were repeated stratified by age (analysed in quartiles), sex, quartiles of total number of consultations (defined as unique dates with face-to-face contact with the practice) and number of comorbidities (Charlson index [21] categorized 0, 1, 2, 3, ≥4).

We quantified the absolute risk of adverse events for each level of a potential prognostic factor in terms of events per 10 000 person-years with accompanying 95% CIs. We fitted age- and sex-adjusted Cox proportional hazards regression models to examine the association between potential risk factors and adverse events. The assumption of proportional hazards was tested graphically using Schoenfeld residuals for each factor. If necessary, an interaction of the prognostic factor with time was estimated. Results were presented as hazard ratios with 95% CIs. Statistical analyses were conducted using Stata version 16.1 (StataCorp LLC, College Station, TX, USA). GOLD and Aurum datasets were analysed separately and combined in a two-stage individual patient data (IPD) meta-analysis. Fixed effects models with an inverse variance approach were used to pool estimates, as clinical and methodological homogeneity was expected between datasets. Meta-analysed results are presented in the manuscript, with results for GOLD and Aurum separately available in the supplementary materials (Supplementary Tables S1–S3, available at *Rheumatology* online).

### Sensitivity analysis

In the main analyses, a missing category approach was used for BMI, as BMI data are unlikely to be missing at random (e.g. BMI is less likely to be recorded in an individual of a healthy weight). In a sensitivity analysis, we generated 10 imputed data sets using multiple imputation through chained equations, and the results were compared with non-imputed data. We also examined adverse events associated with individual statins (simvastatin and atorvastatin as others were small in numbers).

## Results

There were 13 945 people with gout in the two databases (2439 in CPRD GOLD and 11 506 in Aurum) (Table 1). Their mean age was 63.9 (s.d. 14.7) years, and more than two-thirds (78.2%) were male. Background characteristics were fairly similar between both databases. The median time from colchicine initiation to adverse event varied from 21 days [interquartile range (IQR) 21–35] for bone marrow suppression to 61 days (IQR 38–92) for myalgia in CPRD GOLD, and from 63 days (IQR 28–84) for diarrhoea to 70 days (IQR 53–98) for myalgia in CPRD Aurum (Supplementary Table S4, available at *Rheumatology* online).

**Table 1.** Background characteristics of included patients in analysis

Sample characteristics	Combined (%)	CPRD GOLD (%)	CPRD Aurum (%)
Total	13 945	2439	11 506
Mean age at index date (s.d.)	63.9 (14.7)	63.98 (14.6)	63.73 (14.9)
Age quartiles (years)			
Q1 (19–53)	3670 (26.3)	626 (25.7)	3044 (26.5)
Q2 (54–65)	3447 (24.7)	606 (24.8)	2841 (24.7)
Q3 (66–75)	3367 (24.1)	581 (23.8)	2786 24.2)
Q4 (>75)	3461 (24.8)	626 (24.7)	2835 (24.6)
Sex			
Female	3040 (21.8)	545 (22.3)	2495 (21.7)
Male	10 905 (78.2)	1894 (77.7)	9011 (78.3)
BMI			
Normal (18.5–24.9 kg/m^2^)	1958 (14.0)	342 (14.0)	1616 (14.0)
Overweight (25–29.9 kg/m^2^)	4907 (35.2)	904 (37.1)	4003 (34.8)
Obese (≥30 kg/m^2^)	5601 (40.2)	1018 (41.7)	4583 (39.8)
Missing	1479 (10.6)	175 (7.2)	1304 (11.4)
CKD stage			
1–2 (eGFR ≥60 mL/min)	10 154 (72.8)	1701 (69.9)	8453 (73.5)
3 (eGFR 30–45 mL/min)	3179 (22.8)	627 (25.7)	2552 (22.2)
4–5 (eGFR <30 mL/min)	609 (4.4)	108 (4.4)	501 (4.3)
Number of consultations quartiles			
Q1 (0–3)	3312 (23.8)	523 (21.5)	2789 (24.2)
Q2 (4–5)	3430 (24.6)	561 (23.0)	2869 (24.9)
Q3 (6–9)	3538 (25.4)	608 (24.9)	2930 (25.5)
Q4 (≥10)	3665 (26.3)	747 (30.6)	2918 (25.4)
Charlson comorbidity score			
0	4425 (31.7)	750 (30.8)	3675 (31.9)
1	2403 (17.2)	445 (18.2)	1958 (17.0)
2	2324 (16.7)	401 (16.4)	1923 (16.7)
3	1685 (12.1)	293 (12.0)	1392 (12.1)
≥4	3108 (22.3)	550 (22.6)	2558 (22.2)

CPRD: Clinical Practice Research Datalink; CKD: chronic kidney disease; eGFR: estimated glomerular filtration rate.

### Prescription of potentially interacting drugs

Proportions of people with potentially interacting prescriptions issued in the 30-day period prior to the index date are presented in Table 2. One-quarter (0.26, 95% CI 0.25, 0.27) of people were prescribed one or more potentially adverse prescriptions. Statins were the most common prescription (0.21, 95% CI 0.20, 0.22), followed by digoxin (0.05, 95% CI 0.05, 0.05), diltiazem (0.02, 95% CI 0.02, 0.02) and macrolide antibiotics (0.01, 95% CI 0.01, 0.01). Of 2943 people prescribed a statin, 1677 (57.0%) were prescribed simvastatin, 972 (33.0%) atorvastatin, 180 (6.1%) pravastatin, 105 (3.5%) rosuvastatin and 14 (0.4%) fluvastatin. Older age (>75 years), female sex, frequently (≥10) consulting in the last year and Charlson comorbidity index ≥4 were associated with potentially interacting prescriptions (Supplementary Tables S5–S8, available at *Rheumatology* online).

**Table 2.** Proportion of people prescribed colchicine who were also prescribed a potentially interacting medication in the 30-day period prior to the index date by combining CPRD GOLD and Aurum data using two-stage IPD meta-analysis

Frequency and prescriptions of drugs (*N* = 13 945)	Total	Proportion (95% CI)
No. of drugs		
≥1	3625	0.26 (0.253, 0.267)
≥2	590	0.042 (0.039, 0.046)
Specific drugs		
Statins	2943	0.211 (0.204, 0.218)
Fibrates	78	0.006 (0.004, 0.007)
Verapamil	65	0.005 (0.004, 0.006)
Diltiazem	239	0.017 (0.015, 0.019)
Digoxin	697	0.050 (0.046, 0.054)
Amiodarone	93	0.007 (0.005, 0.008)
Oral ketoconazole	21	0.001 (0.001, 0.002)
Macrolide antibiotics	136	0.010 (0.008, 0.011)

CPRD: Clinical Practice Research Datalink; IPD: individual participant data.

The highest rates (per 10 000 person-years) of adverse event reporting were seen for diarrhoea with the prescriptions of fibrates (4322.8, 95% CI 2161.8, 8644.0), myalgia with prescriptions of verapamil (2873.7, 95% CI 404.8, 20 000.0), and diarrhoea with macrolide antibiotics (2855.3, 95% CI 1361.2, 5989.3) (Table 3). There was no overall significantly increased risk of any of the adverse outcomes with the prescription of statins or digoxin (Table 4). The additional sensitivity analysis by individual statins revealed no association of either atorvastatin or simvastatin with any adverse outcome except for atorvastatin with MI [hazard ratio (HR) 2.21, 95% CI 1.14, 4.27] (Supplementary Table S9, available at *Rheumatology* online). However, adverse outcomes could have some potential interaction with medicines, but no obvious patterns of increased risk were observed, and CIs were often very wide.

**Table 3.** Incidence rates of adverse outcomes (per 10 000 person years) by patient characteristics and potentially interacting prescriptions by combining CPRD GOLD and Aurum data using two-stage IPD meta-analysis

Covariates	Diarrhoea	Nausea/vomiting	Neuropathy	Bone marrow suppression	Myalgia	Myocardial infarction
	Rate (95% CI)	Rate (95% CI)	Rate (95% CI)	Rate (95% CI)	Rate (95% CI)	Rate (95% CI)
Age quartiles (years)						
Q1 (19–53)	336.1 (235.0, 480.6)	84.5 (40.3, 177.2)	88.8 (12.5, 630.7)	48.2 (18.1, 128.4)	37.2 (12.0, 115.5)	64.4 (28.9, 143.3)
Q2 (54–65)	575.1 (428.0, 772.8)	176.2 (107.9, 287.6)	84.8 (11.9, 602.1)	45.8 (17.2, 121.9)	239.6 (119.8, 479.1)	195.4 (119.7, 318.9)
Q3 (66–75)	820.4 (639.7, 1052.3)	144.3 (83.8, 248.4)	323.5 (121.4, 862.0)	38.4 (12.4, 119)	128.6 (64.3, 257.1)	169.5 (102.2, 281.2)
Q4 (>75)	1492.5 (1250.7, 1780.9)	452.1 (330.4, 618.8)	92.3 (13, 655.1)	97.7 (48.8, 195.3)	88.3 (42.1, 185.3)	397.1 (283.8, 555.8)
Sex						
Male	600.7 (514.0, 702.1)	128.9 (93.4, 178.0)	164.9 (74.1, 367.0)	56.5 (34.6, 92.2)	115.8 (74.7, 179.5)	200.2 (154.4, 259.5)
Female	1467.4 (1207.4, 1783.4)	502.3 (365.5, 690.3)	101.5 (14.3, 720.8)	45.1 (14.6, 139.9)	90.2 (40.5, 200.9)	189.1 (112, 319.3)
Charlson comorbidity index						
0	377.3 (270.9, 525.5)	104.3 (57.8, 188.4)	144.4 (36.1, 577.5)	19.8 (5.0, 79.3)	141.7 (70.9, 283.3)	147.5 (61.4, 354.3)
1	487.3 (334.2, 710.6)	111.1 (53.0, 233.1)	116.1 (16.4, 824.0)	73.4 (27.6, 195.7)	99.6 (32.1, 308.9)	143.9 (74.9, 276.6)
2	896.4 (681.2, 1179.5)	218.1 (126.7, 375.7)	135.7 (19.1, 963.6)	18.8 (2.7, 133.8)	127.5 (60.8, 267.5)	85.2 (35.5, 204.8)
3	689.4 (479.1, 992.1)	206.2 (103.1, 412.4)	—	142.3 (59.3, 342)	135.8 (51.0, 361.8)	231.5 (120.4, 444.8)
4+	1573.5 (1312.7, 1886.1)	447.3 (322.7, 620.1)	277 (89.3, 858.9)	86.4 (41.2, 181.3)	72.4 (27.2, 193)	537.2 (398.4, 724.3)
BMI						
Normal (18.5–24.9 kg/m^2^)	962.2 (720.7, 1284.6)	291.6 (172.7, 492.3)	—	86.2 (32.4, 229.3)	60.9 (15.2, 243.4)	210.1 (113.0, 390.4)
Overweight (25–29.9 kg/m^2^)	790.5 (642.9, 971.9)	227.9 (158.4, 328.0)	175.7 (56.7, 544.9)	39.8 (16.6, 95.7)	80.3 (41.8, 154.3)	214.8 (147.3, 313.2)
Obese (≥30 kg/m^2^)	749.5 (616.7, 911.0)	163.2 (109.4, 243.5)	203.1 (76.2, 541.1)	61.2 (31.9, 117.7)	165.3 (96.0, 284.8)	218.6 (154.6, 309.1)
Missing	710.2 (467.6, 1078.6)	203.7 (101.9, 407.3)	—	27.3 (3.8, 193.6)	92.8 (23.2, 371)	54.6 (13.7, 218.3)
CKD stage						
1–2 (eGFR ≥60 mL/min)	626 (532.5, 735.8)	162 (120.1, 218.4)	122.9 (46.1, 327.3)	49.3 (28.6, 85)	126.4 (82.4, 193.8)	134.5 (96.1, 188.3)
3 (eGFR 30–45 mL/min)	1189.3 (970.5, 1457.3)	311.2 (210.3, 460.5)	254.8 (82.2, 789.9)	81.8 (36.7, 182)	73.9 (30.8, 177.7)	344.9 (238.1, 499.5)
4–5 (eGFR <30 mL/min)	1355.7 (864.7, 2125.4)	460.8 (219.7, 966.5)	—	—	—	598.4 (311.4, 1150.2)
Adverse prescriptions						
Amiodarone	2134.7 (801.2, 5687.8)	536 (75.5, 3804.7)	—	—	2225.8 (313.5, 16 000.0)	—
Digoxin	1437.6 (971.4, 2127.5)	466.8 (222.6, 979.2)	343 (48.3, 2434.9)	343 (48.3, 2434.9)	345 (48.6, 2448.9)	66.7 (9.4, 473.6)
Diltiazem	1127.8 (506.7, 2510.4)	536.6 (173, 1663.6)	1323.8 (186.5, 9398.1)	—	—	830 (345.5, 1994.0)
Fibrates	4322.8 (2161.8, 8644)	—	—	576.5 (81.2, 4092.4)	572 (80.6, 4061)	588.7 (82.9, 4179.5)
Macrolide antibiotics	2855.3 (1361.2, 5989.3)	729.2 (182.4, 2915.6)	—	—	—	1293.7 (485.6, 3447.0)
Oral ketoconazole	—	—	—	1730.2 (246.6, 12 000)	—	—
Statins	1034.8 (821.4, 1303.7)	341.7 (232.7, 501.9)	196 (49.0, 783.9)	81.3 (33.8, 195.4)	280 (140.0, 559.9)	305.7 (203.1, 460)
Verapamil	1535.7 (384.1, 6140.5)	—	—	—	2873.7 (404.8, 20 000.0)	—

CPRD: Clinical Practice Research Datalink; —: not enough events to calculate estimates; IPD: individual participant data; CKD: chronic kidney disease eGFR: estimated glomerular filtration rate.

**Table 4.** Association of prognostic factors with adverse outcomes, over and above effect of age and sex by combining CPRD GOLD and Aurum data using two-stage IPD meta-analysis

Covariates	Diarrhoea	Nausea/vomiting	Neuropathy	Bone marrow suppression	Myalgia	Myocardial infarction
	HR (95% CI)	HR (95% CI)	HR (95% CI)	HR (95% CI)	HR (95% CI)	HR (95% CI)
Charlson index						
0	1 (Ref)	1 (Ref)	1 (Ref)	1 (Ref)	1 (Ref)	1 (Ref)
1	1.15 (0.69, 1.91)	0.9 (0.34, 2.37)	0.75 (0.07, 8.39)	3.76 (0.68, 20.87)	0.56 (0.15, 2.15)	2.35 (0.52, 10.69)
2	1.97 (1.25, 3.09)	1.34 (0.57, 3.18)	0.93 (0.08, 10.9)	1 (0.09, 11.47)	1.33 (0.43, 4.08)	1.49 (0.31, 7.2)
3	1.32 (0.79, 2.23)	1.32 (0.47, 3.68)	—	4.15 (0.63, 27.46)	0.99 (0.27, 3.61)	16.46 (2.03, 133.76)
4+	2.7 (1.77, 4.11)	1.94 (0.91, 4.14)	1.59 (0.2, 12.9)	4.67 (0.79, 27.74)	0.48 (0.13, 1.78)	4.01 (1.18, 13.59)
BMI						
Normal (18.5–24.9 kg/m^2^)	1 (Ref)	1 (Ref)	1 (Ref)	1 (Ref)	1 (Ref)	1 (Ref)
Overweight (25–29.9 kg/m^2^)	0.87 (0.61, 1.25)	1 (0.53, 1.91)	—	0.52 (0.12, 2.35)	1.74 (0.37, 8.2)	1.2 (0.58, 2.49)
Obese (≥30 kg/m^2^)	0.86 (0.61, 1.23)	0.7 (0.36, 1.37)	1.25 (0.27, 5.72)	0.96 (0.25, 3.7)	2.27 (0.5, 10.2)	1.49 (0.72, 3.08)
Missing	0.86 (0.51, 1.43)	0.97 (0.4, 2.33)	—	0.43 (0.04, 4.19)	1.76 (0.25, 12.63)	0.44 (0.09, 2.09)
CKD stage						
1–2 (eGFR ≥60 mL/min)	1 (Ref)	1 (Ref)	1 (Ref)	1 (Ref)	1 (Ref)	1 (Ref)
3 (eGFR 30–45 mL/min)	1.21 (0.91, 1.61)	0.97 (0.57, 1.65)	2.07 (0.39, 11.13)	0.9 (0.26, 3.08)	0.54 (0.19, 1.53)	1.68 (0.97, 2.92)
4-5 (eGFR <30 mL/min)	1.39 (0.85, 2.28)	1.41 (0.62, 3.22)	—	—	—	3.47 (1.6, 7.5)
Interacting prescriptions						
Amiodarone	3.16 (1.17, 8.54)	2.4 (0.33, 17.35)	—	—	8.27 (1.03, 66.2)	—
Digoxin	1.3 (0.85, 1.97)	1.41 (0.64, 3.13)	2.29 (0.25, 21.11)	—	1.28 (0.16, 10.5)	0.21 (0.03, 1.55)
Diltiazem	1.13 (0.5, 2.53)	2.13 (0.67, 6.8)	8.7 (1.03, 73.38)	—	—	3.8 (1.53, 9.45)
Fibrates	4.58 (2.26, 9.28)	—	—	11.78 (1.55, 89.64)	13.1 (1.7, 101.15)	2.78 (0.39, 20.07)
Macrolide antibiotics	2.4 (1.13, 5.13)	2.35 (0.57, 9.65)	—	—	—	5.37 (1.94, 14.88)
Oral ketoconazole	—	—	—	37.37 (4.91, 284.65)	—	—
Statins	1.1 (0.84, 1.45)	1.49 (0.92, 2.41)	1.27 (0.23, 6.91)	0.78 (0.22, 2.8)	1.77 (0.7, 4.48)	1.35 (0.82, 2.23)
Verapamil	1.05 (0.26, 4.23)	—	—	—	10.88 (1.37, 86.45)	—

Each model was adjusted for age and gender for a given exposure. CPRD: Clinical Practice Research Datalink; —: not enough events to calculate estimates; HR: hazard ratio; CKD: chronic kidney disease; eGFR: estimated glomerular filtration rate; Ref: reference category; IPD: individual participant data.

### Patient-related prognostic factors

Further, the incidence of diarrhoea, nausea and vomiting were generally higher with older age and in females (Table 3). Rates of MI were also higher at older ages. Less clear patterns with age were seen with other adverse outcomes. After adjusting for age and sex, the only significant associations between patient-related prognostic factors and adverse outcomes were Charlson comorbidity index 2 (HR 1.97, 95% CI 1.25, 3.09) and ≥4 (HR 2.70, 95% CI 1.77, 4.11) for diarrhoea; Charlson comorbidity index ≥4 (HR 4.01, 95% CI 1.18, 13.59) for MI (all compared with Charlson index of 0) and CKD stage 4–5 (HR 3.47, 95% CI 1.60, 7.50) compared with stage 1–2 (Table 4). BMI category was not associated with any adverse outcomes in either main or sensitivity analysis (Supplementary Table S10, available at *Rheumatology* online). Myopathy and rhabdomyolysis outcomes were not analysed as there were too few incident occurrences of these outcomes.

## Discussion

This is the first large observational study to investigate prognostic factors for adverse events related to colchicine prophylaxis when initiating allopurinol for gout. Using UK primary care data linked to hospital admission records, we were able to capture rare but potentially serious side effects. We found that females were more likely to develop diarrhoea and nausea/vomiting. Older age was linked to a greater risk of diarrhoea, nausea and vomiting, and myocardial infarction. A quarter of people with gout taking colchicine prophylaxis were already prescribed a medication with the potential to interact with colchicine. In the majority of cases, this was a statin (mainly simvastatin or atorvastatin). Whilst there were some associations of medicines and some adverse outcomes, there were no associations between statins and any adverse outcomes except for atorvastatin with MI. Significant increases in the risk of diarrhoea were seen with amiodarone, fibrates and macrolide antibiotics, neuropathy with diltiazem, bone marrow suppression with fibrates and ketoconazole, myalgia with amiodarone, fibrates and verapamil, and MI with diltiazem and macrolides. A higher comorbidity index was associated with a significantly increased risk of diarrhoea and MI. CKD was significantly associated with an increased risk of MI.

Previous studies have demonstrated the clinical and cost-effectiveness of colchicine prophylaxis when initiating ULT [22, 23]. The risk of adverse events, such as diarrhoea, bone marrow suppression, vomiting or myopathy, from concomitant prescription of colchicine with other medications, including macrolide antibiotics and statins has been highlighted [14, 25–27]. Until now, these observations have been based on case reports, case series, reviews of adverse event reporting systems, or adverse event reporting in individual clinical trials.

We found that concomitant prescriptions of some of these medications are associated with an increased risk of some outcomes, but with no consistent pattern to this association. For example, in our study, fibrates were associated with an increased risk of diarrhoea, bone marrow suppression, myalgia and MI. Anecdotally, the potential interaction of colchicine best recognized by clinicians is that with statins. However, we found no statistically significant increase in adverse events in patients prescribed statins (except for atorvastatin with MI). Statins are used more frequently than fibrates to reduce cholesterol levels and cardiovascular risk. Macrolides are most commonly prescribed as short courses of a few days and hence arguably it would have been more appropriate to ascertain only macrolide prescriptions closer in time to colchicine initiation than the 30-day window employed. However, numbers of macrolide prescriptions in the 10 days preceding colchicine initiation were very small (7 in CPRD GOLD and 36 in Aurum for 1–10 days, 6 in CPRD GOLD and 38 in Aurum for 11–20 days, and 19 in CPRD GOLD and 42 in Aurum for 21–30 days).

Several large randomized controlled trials have shown cardiovascular benefits of colchicine in people with coronary heart disease or post-MI [24, 25, 28, 29]. In these studies, the dose of colchicine used was 0.5 mg daily, which is consistent with that recommended for prophylaxis in gout management guidelines and commonly used in clinical practice [5, 6]. In our study, the risk of MI when commencing colchicine was significantly higher in patients with a history of severe CKD (stage 4 or above), a greater burden of comorbidity, or concomitant prescription of macrolide antibiotics or diltiazem. The increased risk observed in patients with severe CKD is likely to be due to CKD being both a cardiovascular risk factor and associated with traditional cardiovascular risk factors. However, colchicine is relatively contraindicated in people with severe CKD and should be prescribed with caution and at lower doses in that patient group. Furthermore, it is reassuring that no statistically significant increase in MI was seen in those patients with CKD stage 3. It is likely that patients prescribed diltiazem receive it due to underlying hypertension or cardiovascular disease, likely explaining the association seen between its co-prescription with colchicine and risk of MI. However, the increased risk of MI in patients prescribed macrolide antibiotics cannot be explained by these mechanisms, and this has not been observed in previous studies.

Females experienced higher incidence of diarrhoea and nausea/vomiting. The reason for this is not known but it could arise from sex differences in consulting behaviour, symptom reporting or pharmacogenetics, or females with gout being older than males. We did not calculate the risk associated with age and sex as it was a type 2 prognostic study and we decided *a priori* to consider age and sex independent prognostic factors.

The main strengths of our study are the large sample size and the ability to investigate the use of different medicines in clinical practice. Linking primary care consultation and prescription data to hospital records over a period of 20 years allowed us to derive a comprehensive, high-quality dataset. Several limitations are worthy of acknowledgement. First, gout was ascertained according to a clinical diagnosis in primary care rather than classification criteria or synovial fluid microscopy following joint aspiration. However, a coded gout diagnosis in CPRD has a positive predictive value of 90% [18] and these patients were being managed by their general practitioner as though they did have gout, through the prescription of allopurinol. Second, the use of CPRD/HES data permitted us to consider only adverse events severe enough to warrant consultation or result in hospitalization and hence, milder adverse events, such as gastrointestinal symptoms may have been missed. The COLCHICORT trial [30] suggested that co-treatment with a statin could increase the occurrence of mild diarrhoea in people with acute calcium pyrophosphate crystal arthritis. Third, despite the size of our sample and linkage to hospital admissions data, coded occurrences of myopathy and rhabdomyolysis remained rare. This rarity was compounded when stratifying the data, particularly when considering rarely prescribed medications such as oral ketoconazole. Although it was important to interrogate the data and include this medication, there were insufficient numbers of patients even in this large observational cohort to provide clear answers around its risk profile. Fourth, we aimed to study drugs that are commonly prescribed in primary care and did not have access to secondary care prescription data, meaning we were unable to study certain specialist drugs such as targeted anti-cancer therapies and anti-retrovirals. Fifth, analysable data on colchicine dosage were not available as it is most commonly recorded as ‘as directed’ and therefore, no dose-related analyses could be performed. A further caveat is a consequence of the desire of this study to look at a range of medications and outcomes leading to a risk of type I statistical errors due to multiple testing. However, the aim of this study was to provide an overview of the risk of different potential prognostic factors when prescribing colchicine to people with gout rather than to prove causation.

### Conclusion

We found that people were given colchicine prophylaxis despite commonly having preexisting prescriptions for medications with potential to interact with colchicine. Adverse events were more common in people who were older, had more comorbidities and were prescribed certain potentially interacting medications. Reassuringly, the rate of serious adverse events was low, providing reassurance for people with gout and for clinicians. We also found no evidence that the prescription of statins significantly increased the risk of adverse events except atorvastatin being associated with MI.

Future research is needed to determine which patients are at greatest risk of adverse events from prophylaxis and whether the cardiovascular benefits of colchicine reported in randomized controlled trials of people at high risk of cardiovascular events because of a prior history of coronary heart disease also apply to people with gout. Our findings will provide much-needed information about the safety of flare prophylaxis that can inform treatment decisions when initiating allopurinol, directly benefitting people with gout and their clinicians.

## Supplementary Material

keae229_Supplementary_Data

## Data Availability

Data may be obtained from a third party and are not publicly available. The data were obtained from the Clinical Practice Research Datalink (CPRD). CPRD data governance does not allow us to distribute patient data to other parties. Researchers may apply for data access at http://www.CPRD.com. Copyright © (2018), re-used with the permission of The Health & Social Care Information Centre. All rights reserved.
