# Supplementary material for: Prognostic factors for colchicine prophylaxis-related adverse events when initiating allopurinol for gout: retrospective cohort study
Source: Rheumatology (Oxford). 2024 Apr 18;64(3):1147–54. doi: 10.1093/rheumatology/keae229 (PMC11879341; doi:10.1093/rheumatology/keae229)
Supplement: keae229_Supplementary_Data [file keae229_supplementary_data.pdf]

Supplementary Tables

Table S1: Proportion of people by different background categories prescribed colchicine who were also prescribed a potentially interacting medication in the 30-day period prior to the index date separately in CPRD GOLD and Aurum data sets

Data	Group	Outcome	Total patients	Patients with outcome	Proportion
Aurum	overall	drugs	11506	2947	0.2561
Aurum	overall	statins	11506	2401	0.2087
Aurum	overall	fibrates	11506	62	0.0054
Aurum	overall	verapamil	11506	49	0.0043
Aurum	overall	diltiazem	11506	205	0.0178
Aurum	overall	digoxin	11506	552	0.0480
Aurum	overall	amiodarone	11506	72	0.0063
Aurum	overall	ketoconazole	11506	20	0.0017
Aurum	overall	macrolides	11506	107	0.0093
Aurum	male	drugs	9011	2103	0.2334
Aurum	male	statins	9011	1742	0.1933
Aurum	male	fibrates	9011	42	0.0047
Aurum	male	verapamil	9011	26	0.0029
Aurum	male	diltiazem	9011	145	0.0161
Aurum	male	digoxin	9011	347	0.0385
Aurum	male	amiodarone	9011	61	0.0068
Aurum	male	ketoconazole	9011	16	0.0018
Aurum	male	macrolides	9011	68	0.0075
Aurum	female	drugs	2495	844	0.3383
Aurum	female	statins	2495	659	0.2641
Aurum	female	fibrates	2495	20	0.0080
Aurum	female	verapamil	2495	23	0.0092
Aurum	female	diltiazem	2495	60	0.0240
Aurum	female	digoxin	2495	205	0.0822
Aurum	female	amiodarone	2495	11	0.0044
Aurum	female	ketoconazole	2495	--	--
Aurum	female	macrolides	2495	39	0.0156
Aurum	ageq1	drugs	3044	220	0.0723
Aurum	ageq1	statins	3044	181	0.0595
Aurum	ageq1	fibrates	3044	--	--
Aurum	ageq1	verapamil	3044	--	--
Aurum	ageq1	diltiazem	3044	11	0.0036
Aurum	ageq1	digoxin	3044	17	0.0056
Aurum	ageq1	amiodarone	3044	6	0.0020
Aurum	ageq1	ketoconazole	3044	--	--
Aurum	ageq1	macrolides	3044	12	0.0039
Aurum	ageq2	drugs	2841	601	0.2115
Aurum	ageq2	statins	2841	511	0.1799

Aurum	ageq2	fibrates	2841	15	0.0053
Aurum	ageq2	verapamil	2841	11	0.0039
Aurum	ageq2	diltiazem	2841	48	0.0169
Aurum	ageq2	digoxin	2841	70	0.0246
Aurum	ageq2	amiodarone	2841	8	0.0028
Aurum	ageq2	ketoconazole	2841	10	0.0035
Aurum	ageq2	macrolides	2841	20	0.0070
Aurum	ageq3	drugs	2786	993	0.3564
Aurum	ageq3	statins	2786	844	0.3029
Aurum	ageq3	fibrates	2786	25	0.0090
Aurum	ageq3	verapamil	2786	18	0.0065
Aurum	ageq3	diltiazem	2786	79	0.0284
Aurum	ageq3	digoxin	2786	165	0.0592
Aurum	ageq3	amiodarone	2786	25	0.0090
Aurum	ageq3	ketoconazole	2786	--	--
Aurum	ageq3	macrolides	2786	27	0.0097
Aurum	ageq4	drugs	2835	1135	0.4004
Aurum	ageq4	statins	2835	865	0.3051
Aurum	ageq4	fibrates	2835	18	0.0063
Aurum	ageq4	verapamil	2835	18	0.0063
Aurum	ageq4	diltiazem	2835	67	0.0236
Aurum	ageq4	digoxin	2835	300	0.1058
Aurum	ageq4	amiodarone	2835	33	0.0116
Aurum	ageq4	ketoconazole	2835	--	--
Aurum	ageq4	macrolides	2835	48	0.0169
Aurum	cons_0-3	drugs	2789	315	0.1129
Aurum	cons_4-5	drugs	2869	642	0.2238
Aurum	cons_6-9	drugs	2930	863	0.2945
Aurum	cons_10-142	drugs	2918	1121	0.3842
Aurum	cons_0-3	statins	2789	247	0.0886
Aurum	cons_4-5	statins	2869	544	0.1896
Aurum	cons_6-9	statins	2930	719	0.2454
Aurum	cons_10-142	statins	2918	891	0.3053
Aurum	cons_0-3	fibrates	2789	--	--
Aurum	cons_4-5	fibrates	2869	7	0.0024
Aurum	cons_6-9	fibrates	2930	20	0.0068
Aurum	cons_10-142	fibrates	2918	32	0.0110
Aurum	cons_0-3	verapamil	2789	--	--
Aurum	cons_4-5	verapamil	2869	5	0.0017
Aurum	cons_6-9	verapamil	2930	18	0.0061
Aurum	cons_10-142	verapamil	2918	23	0.0079
Aurum	cons_0-3	diltiazem	2789	25	0.0090
Aurum	cons_4-5	diltiazem	2869	30	0.0105

Aurum	cons_6-9	diltiazem	2930	59	0.0201
Aurum	cons_10-142	diltiazem	2918	91	0.0312
Aurum	cons_0-3	digoxin	2789	61	0.0219
Aurum	cons_4-5	digoxin	2869	105	0.0366
Aurum	cons_6-9	digoxin	2930	131	0.0447
Aurum	cons_10-142	digoxin	2918	255	0.0874
Aurum	cons_0-3	amiodarone	2789	7	0.0025
Aurum	cons_4-5	amiodarone	2869	17	0.0059
Aurum	cons_6-9	amiodarone	2930	10	0.0034
Aurum	cons_10-142	amiodarone	2918	38	0.0130
Aurum	cons_0-3	ketoconazole	2789	--	--
Aurum	cons_4-5	ketoconazole	2869	7	0.0024
Aurum	cons_6-9	ketoconazole	2930	--	--
Aurum	cons_10-142	ketoconazole	2918	11	0.0038
Aurum	cons_0-3	macrolides	2789	11	0.0039
Aurum	cons_4-5	macrolides	2869	12	0.0042
Aurum	cons_6-9	macrolides	2930	33	0.0113
Aurum	cons_10-142	macrolides	2918	51	0.0175
Aurum	charl0	drugs	3675	286	0.0778
Aurum	charl1	drugs	1958	449	0.2293
Aurum	charl2	drugs	1923	540	0.2808
Aurum	charl3	drugs	1392	485	0.3484
Aurum	charl4	drugs	2558	1187	0.4640
Aurum	charl0	statins	3675	220	0.0599
Aurum	charl1	statins	1958	353	0.1803
Aurum	charl2	statins	1923	440	0.2288
Aurum	charl3	statins	1392	389	0.2795
Aurum	charl4	statins	2558	999	0.3905
Aurum	charl0	fibrates	3675	--	--
Aurum	charl1	fibrates	1958	5	0.0026
Aurum	charl2	fibrates	1923	16	0.0083
Aurum	charl3	fibrates	1392	15	0.0108
Aurum	charl4	fibrates	2558	23	0.0090
Aurum	charl0	verapamil	3675	6	0.0016
Aurum	charl1	verapamil	1958	10	0.0051
Aurum	charl2	verapamil	1923	5	0.0026
Aurum	charl3	verapamil	1392	8	0.0057
Aurum	charl4	verapamil	2558	20	0.0078
Aurum	charl0	diltiazem	3675	16	0.0044
Aurum	charl1	diltiazem	1958	17	0.0087
Aurum	charl2	diltiazem	1923	43	0.0224
Aurum	charl3	diltiazem	1392	38	0.0273

Aurum	charl4	diltiazem	2558	91	0.0356
Aurum	charl0	digoxin	3675	41	0.0112
Aurum	charl1	digoxin	1958	79	0.0403
Aurum	charl2	digoxin	1923	84	0.0437
Aurum	charl3	digoxin	1392	98	0.0704
Aurum	charl4	digoxin	2558	250	0.0977
Aurum	charl0	amiodarone	3675	--	--
Aurum	charl1	amiodarone	1958	7	0.0036
Aurum	charl2	amiodarone	1923	11	0.0057
Aurum	charl3	amiodarone	1392	7	0.0050
Aurum	charl4	amiodarone	2558	45	0.0176
Aurum	charl0	ketoconazole	3675	--	--
Aurum	charl1	ketoconazole	1958	--	--
Aurum	charl2	ketoconazole	1923	7	0.0036
Aurum	charl3	ketoconazole	1392	--	--
Aurum	charl4	ketoconazole	2558	5	0.0020
Aurum	charl0	macrolides	3675	23	0.0063
Aurum	charl1	macrolides	1958	15	0.0077
Aurum	charl2	macrolides	1923	18	0.0094
Aurum	charl3	macrolides	1392	18	0.0129
Aurum	charl4	macrolides	2558	33	0.0129
Gold	overall	drugs	2439	678	0.2780
Gold	overall	statins	2439	542	0.2222
Gold	overall	fibrates	2439	16	0.0066
Gold	overall	verapamil	2439	16	0.0066
Gold	overall	diltiazem	2439	34	0.0139
Gold	overall	digoxin	2439	145	0.0595
Gold	overall	amiodarone	2439	21	0.0086
Gold	overall	ketoconazole	2439	--	--
Gold	overall	macrolides	2439	29	0.0119
Gold	male	drugs	1894	473	0.2497
Gold	female	drugs	545	205	0.3761
Gold	male	statins	1894	397	0.2096
Gold	female	statins	545	145	0.2661
Gold	male	fibrates	1894	12	0.0063
Gold	female	fibrates	545	--	--
Gold	male	verapamil	1894	9	0.0048
Gold	female	verapamil	545	7	0.0128
Gold	male	diltiazem	1894	24	0.0127
Gold	female	diltiazem	545	10	0.0183
Gold	male	digoxin	1894	84	0.0444
Gold	female	digoxin	545	61	0.1119
Gold	male	amiodarone	1894	19	0.0100
Gold	female	amiodarone	545	--	--
Gold	male	ketoconazole	1894	--	--
Gold	female	ketoconazole	545	0	0.0000

Gold	male	macrolides	1894	15	0.0079
Gold	female	macrolides	545	14	0.0257
Gold	ageq1	drugs	626	44	0.0703
Gold	ageq2	drugs	606	139	0.2294
Gold	ageq3	drugs	581	215	0.3701
Gold	ageq4	drugs	626	280	0.4473
Gold	ageq1	statins	626	33	0.0527
Gold	ageq2	statins	606	120	0.1980
Gold	ageq3	statins	581	182	0.3133
Gold	ageq4	statins	626	207	0.3307
Gold	ageq1	fibrates	626	--	--
Gold	ageq2	fibrates	606	--	--
Gold	ageq3	fibrates	581	5	0.0086
Gold	ageq4	fibrates	626	6	0.0096
Gold	ageq1	verapamil	626	0	0.0000
Gold	ageq2	verapamil	606	5	0.0083
Gold	ageq3	verapamil	581	--	--
Gold	ageq4	verapamil	626	9	0.0144
Gold	ageq1	diltiazem	626	--	--
Gold	ageq2	diltiazem	606	8	0.0132
Gold	ageq3	diltiazem	581	12	0.0207
Gold	ageq4	diltiazem	626	13	0.0208
Gold	ageq1	digoxin	626	5	0.0080
Gold	ageq2	digoxin	606	11	0.0182
Gold	ageq3	digoxin	581	46	0.0792
Gold	ageq4	digoxin	626	83	0.1326
Gold	ageq1	amiodarone	626	--	--
Gold	ageq2	amiodarone	606	--	--
Gold	ageq3	amiodarone	581	8	0.0138
Gold	ageq4	amiodarone	626	8	0.0128
Gold	ageq1	ketoconazole	626	0	0.0000
Gold	ageq2	ketoconazole	606	--	--
Gold	ageq3	ketoconazole	581	0	0.0000
Gold	ageq4	ketoconazole	626	0	0.0000
Gold	ageq1	macrolides	626	5	0.0080
Gold	ageq2	macrolides	606	8	0.0132
Gold	ageq3	macrolides	581	7	0.0120
Gold	ageq4	macrolides	626	9	0.0144
Gold	cons_0-3	drugs	523	55	0.1052
Gold	cons_4-5	drugs	561	141	0.2513
Gold	cons_6-9	drugs	608	180	0.2961
Gold	cons_10-142	drugs	747	302	0.4043
Gold	cons_0-3	statins	523	42	0.0803
Gold	cons_4-5	statins	561	105	0.1872
Gold	cons_6-9	statins	608	148	0.2434

Gold	cons_10-142	statins	747	247	0.3307
Gold	cons_0-3	fibrates	523	--	--
Gold	cons_4-5	fibrates	561	--	--
Gold	cons_6-9	fibrates	608	--	--
Gold	cons_10-142	fibrates	747	10	0.0134
Gold	cons_0-3	verapamil	523	--	--
Gold	cons_4-5	verapamil	561	--	--
Gold	cons_6-9	verapamil	608	--	--
Gold	cons_10-142	verapamil	747	7	0.0094
Gold	cons_0-3	diltiazem	523	--	--
Gold	cons_4-5	diltiazem	561	--	--
Gold	cons_6-9	diltiazem	608	12	0.0197
Gold	cons_10-142	diltiazem	747	16	0.0214
Gold	cons_0-3	digoxin	523	13	0.0249
Gold	cons_4-5	digoxin	561	34	0.0606
Gold	cons_6-9	digoxin	608	30	0.0493
Gold	cons_10-142	digoxin	747	68	0.0910
Gold	cons_0-3	amiodarone	523	--	--
Gold	cons_4-5	amiodarone	561	--	--
Gold	cons_6-9	amiodarone	608	6	0.0099
Gold	cons_10-142	amiodarone	747	9	0.0120
Gold	cons_0-3	ketoconazole	523	0	0.0000
Gold	cons_4-5	ketoconazole	561	0	0.0000
Gold	cons_6-9	ketoconazole	608	0	0.0000
Gold	cons_10-142	ketoconazole	747	--	--
Gold	cons_0-3	macrolides	523	--	--
Gold	cons_4-5	macrolides	561	10	0.0178
Gold	cons_6-9	macrolides	608	7	0.0115
Gold	cons_10-142	macrolides	747	11	0.0147
Gold	charl0	drugs	750	60	0.0800
Gold	charl1	drugs	445	104	0.2337
Gold	charl2	drugs	401	112	0.2793
Gold	charl3	drugs	293	115	0.3925
Gold	charl4	drugs	550	287	0.5218
Gold	charl0	statins	750	48	0.0640
Gold	charl1	statins	445	85	0.1910
Gold	charl2	statins	401	85	0.2120
Gold	charl3	statins	293	92	0.3140
Gold	charl4	statins	550	232	0.4218

Gold	charl0	fibrates	750	--	--
Gold	charl1	fibrates	445	--	--
Gold	charl2	fibrates	401	--	--
Gold	charl3	fibrates	293	--	--
Gold	charl4	fibrates	550	8	0.0145
Gold	charl0	verapamil	750	--	--
Gold	charl1	verapamil	445	--	--
Gold	charl2	verapamil	401	--	--
Gold	charl3	verapamil	293	--	--
Gold	charl4	verapamil	550	8	0.0145
Gold	charl0	diltiazem	750	--	--
Gold	charl1	diltiazem	445	--	--
Gold	charl2	diltiazem	401	5	0.0125
Gold	charl3	diltiazem	293	6	0.0205
Gold	charl4	diltiazem	550	15	0.0273
Gold	charl0	digoxin	750	5	0.0067
Gold	charl1	digoxin	445	14	0.0315
Gold	charl2	digoxin	401	23	0.0574
Gold	charl3	digoxin	293	30	0.1024
Gold	charl4	digoxin	550	73	0.1327
Gold	charl0	amiodarone	750	0	0.0000
Gold	charl1	amiodarone	445	--	--
Gold	charl2	amiodarone	401	--	--
Gold	charl3	amiodarone	293	--	--
Gold	charl4	amiodarone	550	14	0.0255
Gold	charl0	ketoconazole	750	0	0.0000
Gold	charl1	ketoconazole	445	--	--
Gold	charl2	ketoconazole	401	0	0.0000
Gold	charl3	ketoconazole	293	0	0.0000
Gold	charl4	ketoconazole	550	0	0.0000
Gold	charl0	macrolides	750	--	--
Gold	charl1	macrolides	445	6	0.0135
Gold	charl2	macrolides	401	--	--
Gold	charl3	macrolides	293	9	0.0307
Gold	charl4	macrolides	550	7	0.0127
Gold	overall	drugs2	2439	115	0.0472
Gold	male	drugs2	1894	81	0.0428
Gold	female	drugs2	545	34	0.0624
Gold	ageq1	drugs2	626	--	--
Gold	ageq2	drugs2	606	20	0.0330
Gold	ageq3	drugs2	581	43	0.0740
Gold	ageq4	drugs2	626	49	0.0783
Gold	cons_0-3	drugs2	523	9	0.0172
Gold	cons_4-5	drugs2	561	18	0.0321
Gold	cons_6-9	drugs2	608	24	0.0395

Gold	cons_10-142	drugs2	747	64	0.0857
Gold	charl0	drugs2	750	--	--
Gold	charl1	drugs2	445	10	0.0225
Gold	charl2	drugs2	401	12	0.0299
Gold	charl3	drugs2	293	25	0.0853
Gold	charl4	drugs2	550	64	0.1164
Aurum	overall	drugs2	11506	475	0.0413
Aurum	male	drugs2	9011	315	0.0350
Aurum	female	drugs2	2495	160	0.0641
Aurum	ageq1	drugs2	3044	15	0.0049
Aurum	ageq2	drugs2	2841	82	0.0289
Aurum	ageq3	drugs2	2786	178	0.0639
Aurum	ageq4	drugs2	2835	200	0.0705
Aurum	cons_0-3	drugs2	2789	40	0.0143
Aurum	cons_4-5	drugs2	2869	73	0.0254
Aurum	cons_6-9	drugs2	2930	119	0.0406
Aurum	cons_10-142	drugs2	2918	243	0.0833
Aurum	charl0	drugs2	3675	25	0.0068
Aurum	charl1	drugs2	1958	38	0.0194
Aurum	charl2	drugs2	1923	77	0.0400
Aurum	charl3	drugs2	1392	81	0.0582
Aurum	charl4	drugs2	2558	254	0.0993

--: values were masked due to less than 5

Table S2: Incidence rates of adverse outcomes (per 10,000 person years) by patient characteristics and potentially interacting prescriptions separately in CPRD GOLD and Aurum data sets

**Data**	**Outcome**	**Covariate**	**Covariate category**	**No. events**	**Person-year**	**Rate**	**95% Lower limit**	**95% Upper limit**
Gold	diarrhoea	age quartiles	q1	7	0.0112	624.18	297.57	1309.28
Gold	diarrhoea	age quartiles	q2	16	0.0117	1372.67	840.94	2240.61
Gold	diarrhoea	age quartiles	q3	23	0.0122	1885.77	1253.15	2837.77
Gold	diarrhoea	age quartiles	q4	27	0.0105	2559.50	1755.26	3732.23
Gold	diarrhoea	gender	male	44	0.0360	1222.81	909.99	1643.17
Gold	diarrhoea	gender	female	29	0.0096	3010.27	2091.90	4331.82
Gold	diarrhoea	charlson comorbidity	char0	13	0.0137	949.42	551.29	1635.08
Gold	diarrhoea	charlson comorbidity	charl1	9	0.0086	1049.90	546.28	2017.83
Gold	diarrhoea	charlson comorbidity	charl2	12	0.0073	1652.53	938.49	2909.84
Gold	diarrhoea	charlson comorbidity	charl3	7	0.0055	1261.97	601.62	2647.11
Gold	diarrhoea	charlson comorbidity	charl4+	32	0.0105	3035.13	2146.37	4291.91
Gold	diarrhoea	bmi cat	normal	9	0.0062	1446.79	752.79	2780.60
Gold	diarrhoea	bmi cat	overweight	28	0.0169	1656.27	1143.59	2398.80
Gold	diarrhoea	bmi cat	obese	29	0.0194	1493.44	1037.83	2149.08
Gold	diarrhoea	bmi cat	missing	7	0.0031	2278.54	1086.26	4779.48
Gold	diarrhoea	ckd stage	stage 1/2	45	0.0321	1399.70	1045.10	1874.70
Gold	diarrhoea	ckd stage	stage 3	23	0.0116	1982.80	1317.60	2983.80
Gold	diarrhoea	ckd stage	stage 4/5	5	0.0019	2677.40	1114.40	6432.50
Gold	diarrhoea	digoxin	digoxin	6	0.0029	2102.97	944.78	4680.95
Gold	diarrhoea	diltiazem	diltiazem	--	--	2749.34	687.60	10993.07
Gold	diarrhoea	fibrates	fibrates	--	--	8345.40	2691.60	25875.50
Gold	diarrhoea	macrolides	macrolides	--	--	6822.90	2200.50	21154.70
Gold	diarrhoea	statins	statins	21	0.0101	2081.00	1356.80	3191.60
Gold	diarrhoea	verapamil	verapamil	--	--	2950.32	415.59	20944.55
Gold	diarrhoea	amiodarone	amiodarone	0	0.0000	0.00	0.00	0.00
Gold	diarrhoea	ketoconazole	ketoconazole	0	0.0000	0.00	0.00	0.00
Gold	nausea and vomiting	age quartiles	q1	0	0.0000	0.00	0.00	0.00
Gold	nausea and vomiting	age quartiles	q2	--	--	254.85	82.19	790.18
Gold	nausea and vomiting	age quartiles	q3	--	--	161.77	40.46	646.82
Gold	nausea and vomiting	age quartiles	q4	--	--	369.42	138.65	984.30
Gold	nausea and vomiting	gender	male	5	0.0364	137.53	57.24	330.42
Gold	nausea and vomiting	gender	female	--	--	406.08	152.41	1081.97
Gold	nausea and vomiting	charlson comorbidity	char0	--	--	216.45	69.81	671.11
Gold	nausea and vomiting	charlson comorbidity	charl1	--	--	116.23	16.37	825.15
Gold	nausea and vomiting	charlson comorbidity	charl2	--	--	135.95	19.15	965.14

Gold	nausea and vomiting	charlson comorbidity	charl3	0	0.0000	0.00	0.00	0.00
Gold	nausea and vomiting	charlson comorbidity	charl4+	--	--	369.63	138.73	984.85
Gold	nausea and vomiting	bmi cat	normal	--	--	158.57	22.34	1125.70
Gold	nausea and vomiting	bmi cat	overweight	--	--	234.60	88.05	625.07
Gold	nausea and vomiting	bmi cat	obese	--	--	152.43	49.16	472.62
Gold	nausea and vomiting	bmi cat	missing	--	--	315.66	44.47	2240.89
Gold	nausea and vomiting	ckd stage	stage 1/2	6	0.0325	184.58	82.92	410.85
Gold	nausea and vomiting	ckd stage	stage 3	--	--	169.65	42.43	678.34
Gold	nausea and vomiting	ckd stage	stage 4/5	--	--	523.51	73.74	3716.41
Gold	nausea and vomiting	digoxin	digoxin	0	0.0000	0.00	0.00	0.00
Gold	nausea and vomiting	diltiazem	diltiazem	0	0.0000	0.00	0.00	0.00
Gold	nausea and vomiting	fibrates	fibrates	0	0.0000	0.00	0.00	0.00
Gold	nausea and vomiting	macrolides	macrolides	0	0.0000	0.00	0.00	0.00
Gold	nausea and vomiting	statins	statins	--	--	392.00	147.13	1044.46
Gold	nausea and vomiting	verapamil	verapamil	0	0.0000	0.00	0.00	0.00
Gold	nausea and vomiting	amiodarone	amiodarone	0	0.0000	0.00	0.00	0.00
Gold	nausea and vomiting	ketoconazole	ketoconazole	0	0.0000	0.00	0.00	0.00
Gold	neuropathy	age quartiles	q1	--	--	88.85	12.52	630.75
Gold	neuropathy	age quartiles	q2	--	--	84.82	11.95	602.14
Gold	neuropathy	age quartiles	q3	--	--	323.52	121.42	862.00
Gold	neuropathy	age quartiles	q4	--	--	92.29	13.00	655.15
Gold	neuropathy	gender	male	6	0.0364	164.86	74.06	366.95
Gold	neuropathy	gender	female	--	--	101.53	14.30	720.76
Gold	neuropathy	charlson comorbidity	chal0	--	--	144.43	36.12	577.51
Gold	neuropathy	charlson comorbidity	charl1	--	--	116.07	16.35	823.97
Gold	neuropathy	charlson comorbidity	charl2	--	--	135.74	19.12	963.59
Gold	neuropathy	charlson comorbidity	charl3	0	0.0000	0.00	0.00	0.00
Gold	neuropathy	charlson comorbidity	charl4+	--	--	277.01	89.34	858.87
Gold	neuropathy	bmi cat	normal	0	0.0000	0.00	0.00	0.00
Gold	neuropathy	bmi cat	overweight	--	--	175.75	56.68	544.92
Gold	neuropathy	bmi cat	obese	--	--	203.08	76.22	541.08
Gold	neuropathy	bmi cat	missing	0	0.0000	0.00	0.00	0.00
Gold	neuropathy	ckd stage	stage 1/2	--	--	122.86	46.11	327.34
Gold	neuropathy	ckd stage	stage 3	--	--	254.75	82.16	789.88
Gold	neuropathy	ckd stage	stage 4/5	0	0.0000	0.00	0.00	0.00
Gold	neuropathy	digoxin	digoxin	--	--	342.99	48.32	2434.91
Gold	neuropathy	diltiazem	diltiazem	--	--	1323.85	186.48	9398.10
Gold	neuropathy	fibrates	fibrates	0	0.0000	0.00	0.00	0.00
Gold	neuropathy	macrolides	macrolides	0	0.0000	0.00	0.00	0.00
Gold	neuropathy	statins	statins	--	--	196.04	49.03	783.87
Gold	neuropathy	verapamil	verapamil	0	0.0000	0.00	0.00	0.00
Gold	neuropathy	amiodarone	amiodarone	0	0.0000	0.00	0.00	0.00

Gold	neuropathy	ketoconazole	ketoconazole	0	0.0000	0.00	0.00	0.00
Gold	bone_supp	age quartiles	q1	0	0.0000	0.00	0.00	0.00
Gold	bone_supp	age quartiles	q2	--	--	84.81	11.95	602.10
Gold	bone_supp	age quartiles	q3	0	0.0000	0.00	0.00	0.00
Gold	bone_supp	age quartiles	q4	--	--	184.64	46.18	738.26
Gold	bone_supp	gender	male	--	--	82.39	26.57	255.44
Gold	bone_supp	gender	female	0	0.0000	0.00	0.00	0.00
Gold	bone_supp	charlson comorbidity	char0	0	0.0000	0.00	0.00	0.00
Gold	bone_supp	charlson comorbidity	charl1	0	0.0000	0.00	0.00	0.00
Gold	bone_supp	charlson comorbidity	charl2	0	0.0000	0.00	0.00	0.00
Gold	bone_supp	charlson comorbidity	charl3	--	--	358.40	89.64	1433.06
Gold	bone_supp	charlson comorbidity	charl4+	--	--	92.25	12.99	654.86
Gold	bone_supp	bmi cat	normal	--	--	158.49	22.33	1100.00
Gold	bone_supp	bmi cat	overweight	--	--	58.54	8.25	415.57
Gold	bone_supp	bmi cat	obese	--	--	50.75	7.15	360.28
Gold	bone_supp	bmi cat	missing	0	0.0000	0.00	0.00	0.00
Gold	bone_supp	ckd stage	stage 1/2	--	--	30.71	4.33	218.02
Gold	bone_supp	ckd stage	stage 3	--	--	169.59	42.41	678.09
Gold	bone_supp	ckd stage	stage 4/5	0	0.0000	0.00	0.00	0.00
Gold	bone_supp	digoxin	digoxin	--	--	342.99	48.32	2434.91
Gold	bone_supp	diltiazem	diltiazem	0	0.0000	0.00	0.00	0.00
Gold	bone_supp	fibrates	fibrates	0	0.0000	0.00	0.00	0.00
Gold	bone_supp	macrolides	macrolides	0	0.0000	0.00	0.00	0.00
Gold	bone_supp	statins	statins	--	--	196.08	49.04	784.00
Gold	bone_supp	verapamil	verapamil	0	0.0000	0.00	0.00	0.00
Gold	bone_supp	amiodarone	amiodarone	0	0.0000	0.00	0.00	0.00
Gold	bone_supp	ketoconazole	ketoconazole	0	0.0000	0.00	0.00	0.00
Gold	myalgia	age quartiles	q1	--	--	88.85	12.52	630.75
Gold	myalgia	age quartiles	q2	6	0.0118	510.08	229.16	1135.37
Gold	myalgia	age quartiles	q3	--	--	323.26	121.33	861.29
Gold	myalgia	age quartiles	q4	--	--	184.86	46.23	739.14
Gold	myalgia	gender	male	11	0.0364	302.38	167.46	546.02
Gold	myalgia	gender	female	--	--	203.39	50.87	813.23
Gold	myalgia	charlson comorbidity	charl0	5	0.0138	361.47	150.45	868.44
Gold	myalgia	charlson comorbidity	charl1	--	--	232.11	58.05	928.08
Gold	myalgia	charlson comorbidity	charl2	--	--	271.47	67.89	1085.46
Gold	myalgia	charlson comorbidity	charl3	--	--	360.08	90.06	1439.77
Gold	myalgia	charlson comorbidity	charl4+	--	--	184.49	46.14	737.68
Gold	myalgia	bmi cat	normal	--	--	158.49	22.33	1125.11
Gold	myalgia	bmi cat	overweight	--	--	175.79	56.70	545.04

Gold	myalgia	bmi cat	obese	8	0.0197	406.77	203.42	813.37
Gold	myalgia	bmi cat	missing	--	--	315.66	44.47	2240.89
Gold	myalgia	ckd stage	stage 1/2	11	0.0325	338.42	187.42	611.09
Gold	myalgia	ckd stage	stage 3	--	--	169.53	42.40	677.87
Gold	myalgia	ckd stage	stage 4/5	0	0.0000	0.00	0.00	0.00
Gold	myalgia	digoxin	digoxin	--	--	344.97	48.59	2448.94
Gold	myalgia	diltiazem	diltiazem	0	0.0000	0.00	0.00	0.00
Gold	myalgia	fibrates	fibrates	0	0.0000	0.00	0.00	0.00
Gold	myalgia	macrolides	macrolides	0	0.0000	0.00	0.00	0.00
Gold	myalgia	statins	statins	6	0.0102	588.77	264.51	1310.52
Gold	myalgia	verapamil	verapamil	--	--	2873.72	404.80	20400.75
Gold	myalgia	amiodarone	amiodarone	--	--	2225.78	313.53	15800.95
Gold	myalgia	ketoconazole	ketoconazole	0	0.0000	0.00	0.00	0.00
Gold	mi	age quartiles	q1	--	--	89.01	12.54	631.87
Gold	mi	age quartiles	q2	5	0.0118	425.51	177.11	1022.30
Gold	mi	age quartiles	q3	--	--	243.10	78.40	753.73
Gold	mi	age quartiles	q4	--	--	277.18	89.40	859.42
Gold	mi	gender	male	9	0.0363	247.76	128.91	476.17
Gold	mi	gender	female	--	--	305.38	98.49	946.84
Gold	mi	charlson comorbidity	charl0	--	--	289.63	108.70	771.69
Gold	mi	charlson comorbidity	charl1	--	--	116.46	16.41	826.78
Gold	mi	charlson comorbidity	charl2	--	--	135.74	19.12	963.59
Gold	mi	charlson comorbidity	charl3	0	0.0000	0.00	0.00	0.00
Gold	mi	charlson comorbidity	charl4+	6	0.0108	555.50	249.56	1236.47
Gold	mi	bmi cat	normal	--	--	319.08	79.80	1275.82
Gold	mi	bmi cat	overweight	5	0.0170	293.50	122.16	705.15
Gold	mi	bmi cat	obese	5	0.0197	254.09	105.76	610.46
Gold	mi	bmi cat	missing	0	0.0000	0.00	0.00	0.00
Gold	mi	ckd stage	stage 1/2	8	0.0325	246.30	123.18	492.51
Gold	mi	ckd stage	stage 3	--	--	254.85	82.19	790.18
Gold	mi	ckd stage	stage 4/5	--	--	526.98	74.23	3741.07
Gold	mi	digoxin	digoxin	0	0.0000	0.00	0.00	0.00
Gold	mi	diltiazem	diltiazem	--	--	1326.25	186.82	9415.16
Gold	mi	fibrates	fibrates	0	0.0000	0.00	0.00	0.00
Gold	mi	macrolides	macrolides	--	--	2154.87	303.54	15297.56
Gold	mi	statins	statins	--	--	392.66	147.37	1046.20
Gold	mi	verapamil	verapamil	0	0.0000	0.00	0.00	0.00
Gold	mi	amiodarone	amiodarone	0	0.0000	0.00	0.00	0.00
Gold	mi	ketoconazole	ketoconazole	0	0.0000	0.00	0.00	0.00
Aurum	diarrhoea	age quartiles	q1	23	0.0826	278.33	184.96	418.85
Aurum	diarrhoea	age quartiles	q2	28	0.0800	349.82	241.54	506.65
Aurum	diarrhoea	age quartiles	q3	39	0.0777	502.21	366.93	687.37
Aurum	diarrhoea	age quartiles	q4	96	0.0749	1282.37	1049.87	1566.35

Aurum	diarrhoea	gender	male	114	0.2497	456.63	380.05	548.63
Aurum	diarrhoea	gender	female	72	0.0655	1098.63	872.04	1384.10
Aurum	diarrhoea	charlson comorbidity	char0	22	0.1006	218.69	144.00	332.13
Aurum	diarrhoea	charlson comorbidity	charl1	18	0.0542	331.99	209.17	526.94
Aurum	diarrhoea	charlson comorbidity	charl2	39	0.0525	742.58	542.55	1016.36
Aurum	diarrhoea	charlson comorbidity	charl3	22	0.0387	568.80	374.53	863.85
Aurum	diarrhoea	charlson comorbidity	charl4+	85	0.0692	1228.70	993.39	1519.75
Aurum	diarrhoea	bmi cat	normal	37	0.0425	871.32	631.31	1202.59
Aurum	diarrhoea	bmi cat	overweight	62	0.1095	566.01	441.29	725.98
Aurum	diarrhoea	bmi cat	obese	72	0.1268	567.83	450.72	715.37
Aurum	diarrhoea	bmi cat	missing	15	0.0364	412.18	248.49	683.71
Aurum	diarrhoea	ckd stage	stage 1/2	102	0.2324	438.90	361.48	532.90
Aurum	diarrhoea	ckd stage	stage 3	70	0.0696	1005.39	795.42	1270.78
Aurum	diarrhoea	ckd stage	stage 4/5	14	0.0132	1063.16	629.66	1795.12
Aurum	diarrhoea	digoxin	digoxin	19	0.0149	1274.87	813.18	1998.69
Aurum	diarrhoea	diltiazem	diltiazem	--	--	722.34	271.11	1924.60
Aurum	diarrhoea	fibrates	fibrates	5	0.0017	2913.14	1212.53	6998.92
Aurum	diarrhoea	macrolides	macrolides	--	--	1485.66	557.60	3958.41
Aurum	diarrhoea	statins	statins	51	0.0657	776.13	589.85	1021.24
Aurum	diarrhoea	verapamil	verapamil	--	--	799.41	112.61	5675.06
Aurum	diarrhoea	amiodarone	amiodarone	--	--	2134.72	801.20	5687.76
Aurum	diarrhoea	ketoconazole	ketoconazole	0	0.0000	0.00	0.00	0.00
Aurum	nausea and vomiting	age quartiles	q1	7	0.0829	84.48	40.27	177.20
Aurum	nausea and vomiting	age quartiles	q2	13	0.0804	161.78	93.94	278.62
Aurum	nausea and vomiting	age quartiles	q3	11	0.0779	141.28	78.24	255.10
Aurum	nausea and vomiting	age quartiles	q4	35	0.0756	462.71	332.22	644.45
Aurum	nausea and vomiting	gender	male	32	0.2507	127.64	90.26	180.49
Aurum	nausea and vomiting	gender	female	34	0.0660	515.05	368.02	720.82
Aurum	nausea and vomiting	charlson comorbidity	char0	8	0.1008	79.37	39.69	158.70
Aurum	nausea and vomiting	charlson comorbidity	charl1	6	0.0544	110.31	49.56	245.54
Aurum	nausea and vomiting	charlson comorbidity	charl2	12	0.0529	226.90	128.86	399.53
Aurum	nausea and vomiting	charlson comorbidity	charl3	8	0.0388	206.23	103.13	412.38
Aurum	nausea and vomiting	charlson comorbidity	charl4+	32	0.0698	458.13	323.98	647.83
Aurum	nausea and vomiting	bmi cat	normal	13	0.0425	305.57	177.43	526.24
Aurum	nausea and vomiting	bmi cat	overweight	25	0.1102	226.86	153.29	335.74
Aurum	nausea and vomiting	bmi cat	obese	21	0.1274	164.84	107.48	252.82
Aurum	nausea and vomiting	bmi cat	missing	7	0.0366	191.35	91.22	401.37
Aurum	nausea and vomiting	ckd stage	stage 1/2	37	0.2333	158.59	114.90	218.88
Aurum	nausea and vomiting	ckd stage	stage 3	23	0.0701	328.04	217.99	493.65
Aurum	nausea and vomiting	ckd stage	stage 4/5	6	0.0133	451.05	202.64	1003.98

Aurum	nausea and vomiting	digoxin	digoxin	7	0.0150	466.82	222.55	979.20
Aurum	nausea and vomiting	diltiazem	diltiazem	--	--	536.55	173.05	1663.62
Aurum	nausea and vomiting	fibrates	fibrates	0	0.0000	0.00	0.00	0.00
Aurum	nausea and vomiting	macrolides	macrolides	--	--	729.19	182.37	2915.61
Aurum	nausea and vomiting	statins	statins	22	0.0660	333.30	219.46	506.19
Aurum	nausea and vomiting	verapamil	verapamil	0	0.0000	0.00	0.00	0.00
Aurum	nausea and vomiting	amiodarone	amiodarone	--	--	535.95	75.50	3804.75
Aurum	nausea and vomiting	ketoconazole	ketoconazole	0	0.0000	0.00	0.00	0.00
Aurum	neuropathy	age quartiles	q1	0	0.0000	0.00	0.00	0.00
Aurum	neuropathy	age quartiles	q2	0	0.0000	0.00	0.00	0.00
Aurum	neuropathy	age quartiles	q3	0	0.0000	0.00	0.00	0.00
Aurum	neuropathy	age quartiles	q4	0	0.0000	0.00	0.00	0.00
Aurum	neuropathy	gender	male	0	0.0000	0.00	0.00	0.00
Aurum	neuropathy	gender	female	0	0.0000	0.00	0.00	0.00
Aurum	neuropathy	charlson comorbidity	chal0	0	0.0000	0.00	0.00	0.00
Aurum	neuropathy	charlson comorbidity	charl1	0	0.0000	0.00	0.00	0.00
Aurum	neuropathy	charlson comorbidity	charl2	0	0.0000	0.00	0.00	0.00
Aurum	neuropathy	charlson comorbidity	charl3	0	0.0000	0.00	0.00	0.00
Aurum	neuropathy	charlson comorbidity	charl4+	0	0.0000	0.00	0.00	0.00
Aurum	neuropathy	bmi cat	normal	0	0.0000	0.00	0.00	0.00
Aurum	neuropathy	bmi cat	overweight	0	0.0000	0.00	0.00	0.00
Aurum	neuropathy	bmi cat	obese	0	0.0000	0.00	0.00	0.00
Aurum	neuropathy	bmi cat	missing	0	0.0000	0.00	0.00	0.00
Aurum	neuropathy	ckd stage	stage 1/2	0	0.0000	0.00	0.00	0.00
Aurum	neuropathy	ckd stage	stage 3	0	0.0000	0.00	0.00	0.00
Aurum	neuropathy	ckd stage	stage 4/5	0	0.0000	0.00	0.00	0.00
Aurum	neuropathy	digoxin	digoxin	0	0.0000	0.00	0.00	0.00
Aurum	neuropathy	diltiazem	diltiazem	0	0.0000	0.00	0.00	0.00
Aurum	neuropathy	fibrates	fibrates	0	0.0000	0.00	0.00	0.00
Aurum	neuropathy	macrolides	macrolides	0	0.0000	0.00	0.00	0.00
Aurum	neuropathy	statins	statins	0	0.0000	0.00	0.00	0.00
Aurum	neuropathy	verapamil	verapamil	0	0.0000	0.00	0.00	0.00
Aurum	neuropathy	amiodarone	amiodarone	0	0.0000	0.00	0.00	0.00
Aurum	neuropathy	ketoconazole	ketoconazole	0	0.0000	0.00	0.00	0.00
Aurum	bone_supp	age quartiles	q1	--	--	48.20	18.09	128.43
Aurum	bone_supp	age quartiles	q2	--	--	37.26	12.02	115.53
Aurum	bone_supp	age quartiles	q3	--	--	38.38	12.38	118.99
Aurum	bone_supp	age quartiles	q4	6	0.0760	78.99	35.49	175.81
Aurum	bone_supp	gender	male	13	0.2511	51.76	30.06	89.14
Aurum	bone_supp	gender	female	--	--	45.13	14.55	139.92
Aurum	bone_supp	charlson comorbidity	char0	--	--	19.82	4.96	79.25
Aurum	bone_supp	charlson comorbidity	charl1	--	--	73.44	27.56	195.68

Aurum	bone_supp	charlson comorbidity	charl2	--	--	18.85	2.65	133.79
Aurum	bone_supp	charlson comorbidity	charl3	--	--	76.91	24.81	238.48
Aurum	bone_supp	charlson comorbidity	charl4+	6	0.0702	85.47	38.40	190.25
Aurum	bone_supp	bmi cat	normal	--	--	70.19	22.64	217.64
Aurum	bone_supp	bmi cat	overweight	--	--	36.16	13.57	96.35
Aurum	bone_supp	bmi cat	obese	8	0.1276	62.69	31.35	125.35
Aurum	bone_supp	bmi cat	missing	--	--	27.27	3.84	193.62
Aurum	bone_supp	ckd stage	stage 1/2	12	0.2338	51.33	29.15	90.38
Aurum	bone_supp	ckd stage	stage 3	--	--	56.76	21.30	151.24
Aurum	bone_supp	ckd stage	stage 4/5	0	0.0000	0.00	0.00	0.00
Aurum	bone_supp	digoxin	digoxin	0	0.0000	0.00	0.00	0.00
Aurum	bone_supp	diltiazem	diltiazem	0	0.0000	0.00	0.00	0.00
Aurum	bone_supp	fibrates	fibrates	--	--	576.47	81.20	4092.39
Aurum	bone_supp	macrolides	macrolides	0	0.0000	0.00	0.00	0.00
Aurum	bone_supp	statins	statins	--	--	45.23	14.59	140.23
Aurum	bone_supp	verapamil	verapamil	0	0.0000	0.00	0.00	0.00
Aurum	bone_supp	amiodarone	amiodarone	0	0.0000	0.00	0.00	0.00
Aurum	bone_supp	ketoconazole	ketoconazole	--	--	1730.22	243.73	12000.00
Aurum	myalgia	age quartiles	q1	--	--	24.11	6.03	96.38
Aurum	myalgia	age quartiles	q2	--	--	24.84	6.21	99.32
Aurum	myalgia	age quartiles	q3	--	--	51.15	19.20	136.29
Aurum	myalgia	age quartiles	q4	5	0.0761	65.73	27.36	157.92
Aurum	myalgia	gender	male	9	0.2512	35.83	18.64	68.86
Aurum	myalgia	gender	female	--	--	60.12	22.56	160.17
Aurum	myalgia	charlson comorbidity	charl0	--	--	29.75	9.59	92.23
Aurum	myalgia	charlson comorbidity	charl1	--	--	18.35	2.59	130.28
Aurum	myalgia	charlson comorbidity	charl2	5	0.0530	94.25	39.23	226.44
Aurum	myalgia	charlson comorbidity	charl3	--	--	51.22	12.81	204.80
Aurum	myalgia	charlson comorbidity	charl4+	--	--	28.45	7.11	113.74
Aurum	myalgia	bmi cat	normal	--	--	23.38	3.29	165.95
Aurum	myalgia	bmi cat	overweight	6	0.1106	54.23	24.37	120.72
Aurum	myalgia	bmi cat	obese	5	0.1277	39.16	16.30	94.09
Aurum	myalgia	bmi cat	missing	--	--	27.27	3.84	193.62
Aurum	myalgia	ckd stage	stage 1/2	10	0.2338	42.77	23.01	79.48
Aurum	myalgia	ckd stage	stage 3	--	--	42.53	13.72	131.87
Aurum	myalgia	ckd stage	stage 4/5	0	0.0000	0.00	0.00	0.00
Aurum	myalgia	digoxin	digoxin	0	0.0000	0.00	0.00	0.00
Aurum	myalgia	diltiazem	diltiazem	0	0.0000	0.00	0.00	0.00
Aurum	myalgia	fibrates	fibrates	--	--	572.04	80.58	4060.98
Aurum	myalgia	macrolides	macrolides	0	0.0000	0.00	0.00	0.00
Aurum	myalgia	statins	statins	--	--	30.12	7.53	120.42

Aurum	myalgia	verapamil	verapamil	0	0.0000	0.00	0.00	0.00
Aurum	myalgia	amiodarone	amiodarone	0	0.0000	0.00	0.00	0.00
Aurum	myalgia	ketoconazole	ketoconazole	0	0.0000	0.00	0.00	0.00
Aurum	mi	age quartiles	q1	5	0.0829	60.35	25.12	144.98
Aurum	mi	age quartiles	q2	11	0.0802	137.18	75.97	247.70
Aurum	mi	age quartiles	q3	12	0.0775	154.94	87.99	272.82
Aurum	mi	age quartiles	q4	31	0.0754	411.21	289.19	584.72
Aurum	mi	gender	male	48	0.2496	192.31	144.93	255.20
Aurum	mi	gender	female	11	0.0663	165.94	91.90	299.64
Aurum	mi	charlson comorbidity	charl0	--	--	9.91	1.40	70.36
Aurum	mi	charlson comorbidity	charl1	8	0.0541	147.80	73.91	295.54
Aurum	mi	charlson comorbidity	charl2	--	--	75.87	28.47	202.14
Aurum	mi	charlson comorbidity	charl3	9	0.0389	231.45	120.43	444.83
Aurum	mi	charlson comorbidity	charl4+	37	0.0693	534.26	387.09	737.38
Aurum	mi	bmi cat	normal	8	0.0423	189.24	94.64	378.40
Aurum	mi	bmi cat	overweight	22	0.1100	200.08	131.74	303.86
Aurum	mi	bmi cat	obese	27	0.1270	212.56	145.77	309.96
Aurum	mi	bmi cat	missing	--	--	54.61	13.66	218.34
Aurum	mi	ckd stage	stage 1/2	26	0.2328	111.68	76.04	164.02
Aurum	mi	ckd stage	stage 3	25	0.0699	357.60	241.63	529.22
Aurum	mi	ckd stage	stage 4/5	8	0.0132	608.03	304.07	1215.82
Aurum	mi	digoxin	digoxin	--	--	66.72	9.40	473.62
Aurum	mi	diltiazem	diltiazem	--	--	738.18	277.05	1966.81
Aurum	mi	fibrates	fibrates	--	--	588.73	82.93	4179.46
Aurum	mi	macrolides	macrolides	--	--	1091.38	351.99	3383.91
Aurum	mi	statins	statins	19	0.0655	290.00	184.98	454.65
Aurum	mi	verapamil	verapamil	0	0.0000	0.00	0.00	0.00
Aurum	mi	amiodarone	amiodarone	0	0.0000	0.00	0.00	0.00
Aurum	mi	ketoconazole	ketoconazole	0	0.0000	0.00	0.00	0.00

--: values were masked due to less than 5

Table S3: Association of prognostic factors with adverse outcomes, over and above effect of age and sex separately in CPRD GOLD and Aurum data sets

Data	Outcome	Covariate	Covariate category	HR	95% Lower limit	95% Upper limit	P-value
Gold	diarrhoea	charlson comorbidity	charl1	0.93	0.40	2.20	0.875
Gold	diarrhoea	charlson comorbidity	charl2	1.27	0.57	2.86	0.559
Gold	diarrhoea	charlson comorbidity	charl3	0.79	0.30	2.07	0.631
Gold	diarrhoea	charlson comorbidity	charl4+	1.91	0.93	3.92	0.080
Gold	diarrhoea	bmi cat	overweight	1.25	0.59	2.66	0.559
Gold	diarrhoea	bmi cat	obese	1.11	0.52	2.36	0.793
Gold	diarrhoea	bmi cat	missing	1.90	0.70	5.13	0.207
Gold	diarrhoea	ckd stage	stage 3	0.84	0.49	1.44	0.520
Gold	diarrhoea	ckd stage	stage4/5	1.15	0.45	2.96	0.770
Gold	diarrhoea	digoxin	digoxin	0.84	0.36	1.96	0.680
Gold	diarrhoea	diltiazem	diltiazem	1.40	0.34	5.73	0.638
Gold	diarrhoea	fibrates	fibrates	5.37	1.68	17.11	0.005
Gold	diarrhoea	macrolides	macrolides	3.42	1.06	11.01	0.040
Gold	diarrhoea	statins	statins	1.11	0.67	1.86	0.682
Gold	diarrhoea	verapamil	verapamil	1.16	0.16	8.39	0.886
Gold	diarrhoea	amiodarone	amiodarone	--	--	--	--
Gold	diarrhoea	ketoconazole	ketoconazole	--	--	--	--
Gold	nausea and vomiting	charlson comorbidity	charl1	0.36	0.04	3.56	0.382
Gold	nausea and vomiting	charlson comorbidity	charl2	0.32	0.03	3.26	0.334
Gold	nausea and vomiting	charlson comorbidity	charl3	--	--	--	--
Gold	nausea and vomiting	charlson comorbidity	charl4+	0.58	0.11	3.17	0.530
Gold	nausea and vomiting	bmi cat	overweight	1.74	0.19	15.67	0.620
Gold	nausea and vomiting	bmi cat	obese	1.17	0.12	11.61	0.890
Gold	nausea and vomiting	bmi cat	missing	2.71	0.17	44.02	0.483
Gold	nausea and vomiting	ckd stage	stage 3	0.43	0.08	2.33	0.329
Gold	nausea and vomiting	ckd stage	stage4/5	1.37	0.16	11.94	0.778
Gold	nausea and vomiting	digoxin	digoxin	--	--	--	--
Gold	nausea and vomiting	diltiazem	diltiazem	--	--	--	--
Gold	nausea and vomiting	fibrates	fibrates	--	--	--	--
Gold	nausea and vomiting	macrolides	macrolides	--	--	--	--
Gold	nausea and vomiting	statins	statins	2.09	0.55	7.90	0.277
Gold	nausea and vomiting	verapamil	verapamil	--	--	--	--
Gold	nausea and vomiting	amiodarone	amiodarone	--	--	--	--
Gold	nausea and vomiting	ketoconazole	ketoconazole	--	--	--	--
Gold	neuropathy	charlson comorbidity	charl1	0.75	0.07	8.39	0.812
Gold	neuropathy	charlson comorbidity	charl2	0.93	0.08	10.90	0.957
Gold	neuropathy	charlson comorbidity	charl3	--	--	--	--
Gold	neuropathy	charlson comorbidity	charl4+	1.59	0.20	12.90	0.662
Gold	neuropathy	bmi cat	overweight	--	--	--	--
Gold	neuropathy	bmi cat	obese	1.25	0.27	5.72	0.772
Gold	neuropathy	bmi cat	missing	--	--	--	--

Gold	neuropathy	ckd stage	stage 3	2.07	0.39	11.13	0.396
Gold	neuropathy	ckd stage	stage4/5	--	--	--	--
Gold	neuropathy	digoxin	digoxin	2.29	0.25	21.11	0.464
Gold	neuropathy	diltiazem	diltiazem	8.70	1.03	73.38	0.047
Gold	neuropathy	fibrates	fibrates	--	--	--	--
Gold	neuropathy	macrolides	macrolides	--	--	--	--
Gold	neuropathy	statins	statins	1.26	0.23	6.91	0.786
Gold	neuropathy	verapamil	verapamil	--	--	--	--
Gold	neuropathy	amiodarone	amiodarone	--	--	--	--
Gold	neuropathy	ketoconazole	ketoconazole	--	--	--	--
Gold	bone_supp	charlson comorbidity	charl1	--	--	--	--
Gold	bone_supp	charlson comorbidity	charl2	--	--	--	--
Gold	bone_supp	charlson comorbidity	charl3	--	--	--	--
Gold	bone_supp	charlson comorbidity	charl4+	--	--	--	--
Gold	bone_supp	bmi cat	overweight	--	--	--	--
Gold	bone_supp	bmi cat	obese	--	--	--	--
Gold	bone_supp	bmi cat	missing	--	--	--	--
Gold	bone_supp	ckd stage	stage 3	--	--	--	--
Gold	bone_supp	ckd stage	stage4/5	--	--	--	--
Gold	bone_supp	digoxin	digoxin	--	--	--	--
Gold	bone_supp	diltiazem	diltiazem	--	--	--	--
Gold	bone_supp	fibrates	fibrates	--	--	--	--
Gold	bone_supp	macrolides	macrolides	--	--	--	--
Gold	bone_supp	statins	statins	--	--	--	--
Gold	bone_supp	verapamil	verapamil	--	--	--	--
Gold	bone_supp	amiodarone	amiodarone	--	--	--	--
Gold	bone_supp	ketoconazole	ketoconazole	--	--	--	--
Gold	myalgia	charlson comorbidity	charl1	0.60	0.12	3.15	0.550
Gold	myalgia	charlson comorbidity	charl2	0.76	0.14	4.07	0.752
Gold	myalgia	charlson comorbidity	charl3	0.98	0.17	5.78	0.983
Gold	myalgia	charlson comorbidity	charl4+	0.46	0.08	2.81	0.403
Gold	myalgia	bmi cat	overweight	1.08	0.11	10.43	0.944
Gold	myalgia	bmi cat	obese	2.56	0.32	20.68	0.378
Gold	myalgia	bmi cat	missing	2.07	0.13	33.79	0.609
Gold	myalgia	ckd stage	stage 3	0.49	0.10	2.41	0.378
Gold	myalgia	ckd stage	stage4/5	--	--	--	--
Gold	myalgia	digoxin	digoxin	1.28	0.16	10.50	0.816
Gold	myalgia	diltiazem	diltiazem	--	--	--	--
Gold	myalgia	fibrates	fibrates	--	--	--	--
Gold	myalgia	macrolides	macrolides	--	--	--	--
Gold	myalgia	statins	statins	3.60	1.10	11.71	0.034
Gold	myalgia	verapamil	verapamil	10.88	1.37	86.45	0.024
Gold	myalgia	amiodarone	amiodarone	8.27	1.03	66.20	0.047
Gold	myalgia	ketoconazole	ketoconazole	--	--	--	--
Gold	mi	charlson comorbidity	charl1	0.35	0.04	3.22	0.357
Gold	mi	charlson comorbidity	charl2	0.36	0.04	3.37	0.368

Gold	mi	charlson comorbidity	charl3	--	--	--	--
Gold	mi	charlson comorbidity	charl4+	1.19	0.26	5.48	0.823
Gold	mi	bmi cat	overweight	0.98	0.19	5.07	0.980
Gold	mi	bmi cat	obese	0.91	0.17	4.79	0.910
Gold	mi	bmi cat	missing	--	--	--	--
Gold	mi	ckd stage	stage 3	0.68	0.16	2.83	0.594
Gold	mi	ckd stage	stage4/5	1.54	0.18	13.05	0.691
Gold	mi	digoxin	digoxin	--	--	--	--
Gold	mi	diltiazem	diltiazem	4.98	0.64	38.99	0.126
Gold	mi	fibrates	fibrates	--	--	--	--
Gold	mi	macrolides	macrolides	7.90	0.97	64.35	0.053
Gold	mi	statins	statins	1.44	0.42	4.94	0.564
Gold	mi	verapamil	verapamil	--	--	--	--
Gold	mi	amiodarone	amiodarone	--	--	--	--
Gold	mi	ketoconazole	ketoconazole	--	--	--	--
Aurum	diarrhoea	charlson comorbidity	charl1	1.28	0.68	2.40	0.437
Aurum	diarrhoea	charlson comorbidity	charl2	2.39	1.39	4.11	0.002
Aurum	diarrhoea	charlson comorbidity	charl3	1.64	0.88	3.04	0.119
Aurum	diarrhoea	charlson comorbidity	charl4+	3.23	1.93	5.41	<0.001
Aurum	diarrhoea	bmi cat	overweight	0.78	0.52	1.18	0.235
Aurum	diarrhoea	bmi cat	obese	0.80	0.54	1.21	0.292
Aurum	diarrhoea	bmi cat	missing	0.64	0.35	1.17	0.143
Aurum	diarrhoea	ckd stage	stage 3	1.39	1.00	1.93	0.052
Aurum	diarrhoea	ckd stage	stage4/5	1.50	0.84	2.66	0.170
Aurum	diarrhoea	digoxin	digoxin	1.49	0.92	2.42	0.104
Aurum	diarrhoea	diltiazem	diltiazem	1.01	0.37	2.72	0.985
Aurum	diarrhoea	fibrates	fibrates	4.18	1.72	10.16	0.002
Aurum	diarrhoea	macrolides	macrolides	1.87	0.69	5.04	0.219
Aurum	diarrhoea	statins	statins	1.10	0.79	1.52	0.575
Aurum	diarrhoea	verapamil	verapamil	0.95	0.13	6.78	0.958
Aurum	diarrhoea	amiodarone	amiodarone	3.16	1.17	8.54	0.023
Aurum	diarrhoea	ketoconazole	ketoconazole	--	--	--	--
Aurum	nausea and vomiting	charlson comorbidity	charl1	1.10	0.38	3.19	0.862
Aurum	nausea and vomiting	charlson comorbidity	charl2	1.69	0.67	4.26	0.268
Aurum	nausea and vomiting	charlson comorbidity	charl3	1.32	0.47	3.68	0.597
Aurum	nausea and vomiting	charlson comorbidity	charl4+	2.62	1.12	6.12	0.026
Aurum	nausea and vomiting	bmi cat	overweight	0.95	0.49	1.87	0.887
Aurum	nausea and vomiting	bmi cat	obese	0.67	0.33	1.35	0.258
Aurum	nausea and vomiting	bmi cat	missing	0.87	0.34	2.18	0.760
Aurum	nausea and vomiting	ckd stage	stage 3	1.06	0.61	1.85	0.831
Aurum	nausea and vomiting	ckd stage	stage4/5	1.42	0.59	3.45	0.437
Aurum	nausea and vomiting	digoxin	digoxin	1.41	0.64	3.13	0.397
Aurum	nausea and vomiting	diltiazem	diltiazem	2.13	0.67	6.80	0.200
Aurum	nausea and vomiting	fibrates	fibrates	--	--	--	--
Aurum	nausea and vomiting	macrolides	macrolides	2.35	0.57	9.65	0.236
Aurum	nausea and vomiting	statins	statins	1.41	0.84	2.37	0.188

Aurum	nausea and vomiting	verapamil	verapamil	--	--	--	--
Aurum	nausea and vomiting	amiodarone	amiodarone	2.40	0.33	17.35	0.386
Aurum	nausea and vomiting	ketoconazole	ketoconazole	--	--	--	--
Aurum	neuropathy	charlson comorbidity	charl1	--	--	--	--
Aurum	neuropathy	charlson comorbidity	charl2	--	--	--	--
Aurum	neuropathy	charlson comorbidity	charl3	--	--	--	--
Aurum	neuropathy	charlson comorbidity	charl4+	--	--	--	--
Aurum	neuropathy	bmi cat	overweight	--	--	--	--
Aurum	neuropathy	bmi cat	obese	--	--	--	--
Aurum	neuropathy	bmi cat	missing	--	--	--	--
Aurum	neuropathy	ckd stage	stage 3	--	--	--	--
Aurum	neuropathy	ckd stage	stage4/5	--	--	--	--
Aurum	neuropathy	digoxin	digoxin	--	--	--	--
Aurum	neuropathy	diltiazem	diltiazem	--	--	--	--
Aurum	neuropathy	fibrates	fibrates	--	--	--	--
Aurum	neuropathy	macrolides	macrolides	--	--	--	--
Aurum	neuropathy	statins	statins	--	--	--	--
Aurum	neuropathy	verapamil	verapamil	--	--	--	--
Aurum	neuropathy	amiodarone	amiodarone	--	--	--	--
Aurum	neuropathy	ketoconazole	ketoconazole	--	--	--	--
Aurum	bone_supp	charlson comorbidity	charl1	3.76	0.68	20.87	0.130
Aurum	bone_supp	charlson comorbidity	charl2	1.00	0.09	11.47	1.000
Aurum	bone_supp	charlson comorbidity	charl3	4.15	0.63	27.46	0.140
Aurum	bone_supp	charlson comorbidity	charl4+	4.67	0.79	27.74	0.090
Aurum	bone_supp	bmi cat	overweight	0.52	0.12	2.35	0.397
Aurum	bone_supp	bmi cat	obese	0.96	0.25	3.70	0.955
Aurum	bone_supp	bmi cat	missing	0.43	0.04	4.19	0.466
Aurum	bone_supp	ckd stage	stage 3	0.90	0.26	3.08	0.869
Aurum	bone_supp	ckd stage	stage4/5	--	--	--	--
Aurum	bone_supp	digoxin	digoxin	--	--	--	--
Aurum	bone_supp	diltiazem	diltiazem	--	--	--	--
Aurum	bone_supp	fibrates	fibrates	11.78	1.55	89.64	0.017
Aurum	bone_supp	macrolides	macrolides	--	--	--	--
Aurum	bone_supp	statins	statins	0.78	0.22	2.80	0.705
Aurum	bone_supp	verapamil	verapamil	--	--	--	--
Aurum	bone_supp	amiodarone	amiodarone	--	--	--	--
Aurum	bone_supp	ketoconazole	ketoconazole	37.37	4.91	284.65	p<0.001
Aurum	myalgia	charlson comorbidity	charl1	0.50	0.05	4.84	0.546
Aurum	myalgia	charlson comorbidity	charl2	2.08	0.46	9.49	0.342
Aurum	myalgia	charlson comorbidity	charl3	0.99	0.15	6.66	0.995
Aurum	myalgia	charlson comorbidity	charl4+	0.49	0.07	3.43	0.473
Aurum	myalgia	bmi cat	overweight	2.65	0.32	22.14	0.369
Aurum	myalgia	bmi cat	obese	1.99	0.23	17.37	0.533
Aurum	myalgia	bmi cat	missing	1.49	0.09	24.20	0.778
Aurum	myalgia	ckd stage	stage 3	0.59	0.15	2.31	0.445
Aurum	myalgia	ckd stage	stage4/5	--	--	--	--

Aurum	myalgia	digoxin	digoxin	--	--	--	--
Aurum	myalgia	diltiazem	diltiazem	--	--	--	--
Aurum	myalgia	fibrates	fibrates	13.10	1.70	101.15	0.014
Aurum	myalgia	macrolides	macrolides	--	--	--	--
Aurum	myalgia	statins	statins	0.54	0.12	2.49	0.433
Aurum	myalgia	verapamil	verapamil	--	--	--	--
Aurum	myalgia	amiodarone	amiodarone	--	--	--	--
Aurum	myalgia	ketoconazole	ketoconazole	--	--	--	--
Aurum	mi	charlson comorbidity	charl1	12.62	1.57	101.45	0.017
Aurum	mi	charlson comorbidity	charl2	5.96	0.66	54.15	0.113
Aurum	mi	charlson comorbidity	charl3	16.46	2.02	133.76	0.009
Aurum	mi	charlson comorbidity	charl4+	34.49	4.52	262.99	0.001
Aurum	mi	bmi cat	overweight	1.26	0.56	2.84	0.580
Aurum	mi	bmi cat	obese	1.68	0.75	3.75	0.208
Aurum	mi	bmi cat	missing	0.44	0.09	2.09	0.303
Aurum	mi	ckd stage	stage 3	1.97	1.08	3.58	0.026
Aurum	mi	ckd stage	stage4/5	3.91	1.71	8.96	0.001
Aurum	mi	digoxin	digoxin	0.21	0.03	1.55	0.128
Aurum	mi	diltiazem	diltiazem	3.56	1.29	9.83	0.014
Aurum	mi	fibrates	fibrates	2.78	0.38	20.07	0.311
Aurum	mi	macrolides	macrolides	4.76	1.48	15.30	0.009
Aurum	mi	statins	statins	1.33	0.77	2.31	0.305
Aurum	mi	verapamil	verapamil	--	--	--	--
Aurum	mi	amiodarone	amiodarone	--	--	--	--
Aurum	mi	ketoconazole	ketoconazole	--	--	--	--

HR: hazard ratio; --: not estimable as there were no events

Table S4: Median time between the therapy initiation and adverse event in the CPRD GOLD and Aurum data sets

**Adverse outcomes**	**CPRD GOLD**	**CPRD Aurum**
	Median (IQR)	Median (IQR)
Diarrhoea	43.0 (16.0-58.0)	63.0 (28.0-84.0)
Nausea/vomiting	35.0 (10.0-38.0)	63.0 (22.0-82.0)
Neuropathy	38.0 (19.0-70.0)	--
Bone marrow suppression	21.0 (21.0-35.0)	66.5 (27.5-102.0)
Myalgia	61.0 (38.0-92.0)	70.0 (53.0-98.0)
Myocardial infarction	41.5 (33.5-50.0)	66.0 (35.0-98.0)

IQR: interquartile range; --: insufficient events to calculate estimate

Table S5: Potentially interacting prescriptions issued in the 30-day period prior to the index date by sex by combining CPRD GOLD and Aurum data using two-stage IPD meta-analysis

	Overall				Male				Female			
	N	Proportion	95% CI		N	Proportion	95% CI		N	Proportion	95% CI	
			LCL	UCL			LCL	UCL			LCL	UCL
Drugs												
≥1 drug	3625	0.2599	0.2526	0.2672	2576	0.2362	0.2282	0.2442	1049	0.3449	0.3281	0.3619
≥2 drugs	590	0.0422	0.0390	0.0456	396	0.0362	0.0328	0.0398	194	0.0636	0.0552	0.0726
Specific drugs												
Statins	2943	0.2110	0.2043	0.2178	2139	0.1961	0.1887	0.2036	804	0.2644	0.2488	0.2802
Fibrates	78	0.0055	0.0044	0.0068	54	0.0049	0.0036	0.0063	24	0.0077	0.0048	0.0112
Verapamil	65	0.0046	0.0035	0.0058	35	0.0031	0.0021	0.0043	30	0.0096	0.0063	0.0135
Diltiazem	239	0.0171	0.0150	0.0193	169	0.0154	0.0132	0.0178	70	0.0228	0.0177	0.0284
Digoxin	697	0.0498	0.0463	0.0535	431	0.0394	0.0359	0.0432	266	0.0870	0.0772	0.0973
Amiodarone	93	0.0066	0.0053	0.0080	80	0.0072	0.0057	0.0089	13	0.0041	0.002	0.0068
Oral ketoconazole	21	0.0014	0.0008	0.0021	*	**	**	**	*	**	**	**
Macrolide antibiotics	136	0.0097	0.0081	0.0114	83	0.0075	0.006	0.0093	53	0.0170	0.0126	0.022

*N<5, **Omitted to mask not to recalculate number; IPD: individual participant data; LCL: lower confidence limit; UCL: upper confidence limit; CI: confidence interval.

Table S6: Potentially interacting prescriptions issued in the 30-day period prior to the index date by age quartile by combining CPRD GOLD and Aurum data using two-stage IPD meta-analysis

	Age quartile 1 (19 – 53 years)				Age quartile 2 (54 – 65 years)				Age quartile 3 (66 – 75 years)				Age quartile 4 (>75 years)			
	N	Proportion	95% CI		N	Proportion	95% CI		N	Proportion	95% CI		N	Proportion	95% CI	
			LCL	UCL			LCL	UCL			LCL	UCL			LCL	UCL
Drugs																
≥1 drug	264	0.0718	0.0636	0.0804	740	0.2145	0.201	0.2284	1208	0.3587	0.3426	0.3750	1415	0.4087	0.3924	0.4252
≥2 drugs	18	0.0047	0.0026	0.0073	102	0.0294	0.0239	0.0353	221	0.0654	0.0573	0.0740	249	0.0717	0.0634	0.0806
Specific drugs																
Statins	224	0.0581	0.0507	0.0659	631	0.1829	0.1701	0.1960	1026	0.3046	0.2892	0.3203	1072	0.3096	0.2943	0.3251
Fibrates	6	0.0014	0.0003	0.0030	18	0.0050	0.0028	0.0078	30	0.0087	0.0058	0.0122	24	0.0067	0.0041	0.0098
Verapamil	*	**	**	**	*	**	0.0023	0.0069	20	0.0057	0.0033	0.0086	27	0.0073	0.0047	0.0106
Diltiazem	12	0.0030	0.0014	0.0052	52	0.0160	0.012	0.0205	91	0.0267	0.0215	0.0325	80	0.0229	0.0181	0.0282
Digoxin	22	0.0057	0.0035	0.0085	81	0.0232	0.0184	0.0286	211	0.0623	0.0543	0.0707	383	0.1103	0.1001	0.1210
Amiodarone	8	0.0019	0.0007	0.0038	11	0.0029	0.0013	0.0051	33	0.0095	0.0064	0.0131	41	0.0116	0.0083	0.0156
Oral ketoconazole	*	**	**	**	11	0.0030	0.0013	0.0052	*	**	**	**	*	**	0.0001	0.0019
Macrolide antibiotics	17	0.0043	0.0023	0.0068	28	0.0078	0.005	0.0111	34	0.0099	0.0067	0.0136	57	0.0163	0.0123	0.0208

*N<5, **Omitted to mask not to recalculate number; IPD: individual participant data; LCL: lower confidence limit; UCL: upper confidence limit; CI: confidence interval.

Table S7: Potentially interacting prescriptions issued in the 30-day period prior to the index date by number of consultations (quartiles) by combining CPRD GOLD and Aurum data using two-stage IPD meta-analysis

	Number of consultations quartile 1 (0 – 3)				Number of consultations quartile 2 (4 – 5)				Number of consultations quartile 3 (6 – 9)				Number of consultations quartile 4 (≥10)			
	N	Proportion	95% CI		N	Proportion	95% CI		N	Proportion	95% CI		N	Proportion	95% CI	
			LCL	UCL			LCL	UCL			LCL	UCL			LCL	UCL
Drugs																
≥1 drug	370	0.1115	0.1010	0.1225	783	0.2281	0.2142	0.2423	1043	0.2947	0.2798	0.3099	1423	0.3882	0.3725	0.4041
≥2 drugs	49	0.0145	0.0106	0.0189	91	0.0262	0.0211	0.0319	143	0.0402	0.034	0.0470	307	0.0836	0.0749	0.0928
Specific drugs																
Statins	289	0.0870	0.0776	0.0969	649	0.1891	0.1761	0.2024	867	0.2449	0.2309	0.2593	1138	0.3104	0.2955	0.3255
Fibrates	*	**	**	**	*	**	0.0010	0.0047	22	0.0060	0.0036	0.0089	42	0.0113	0.0081	0.0150
Verapamil	5	0.0011	0.0001	0.0028	9	0.0021	0.0007	0.0041	21	0.0057	0.0034	0.0086	30	0.0080	0.0053	0.0112
Diltiazem	28	0.0082	0.0053	0.0116	33	0.0093	0.0063	0.0129	71	0.0199	0.0155	0.0248	107	0.0289	0.0237	0.0346
Digoxin	74	0.0221	0.0173	0.0274	139	0.0399	0.0336	0.0467	161	0.0453	0.0387	0.0524	323	0.0880	0.079	0.0974
Amiodarone	9	0.0024	0.0009	0.0046	21	0.0059	0.0035	0.0088	16	0.0041	0.0021	0.0065	57	0.0127	0.0093	0.0166
Oral ketoconazole	*	**	**	**	7	0.0016	0.0004	0.0034	*	**	**	**	12	0.0031	0.0014	0.0052
Macrolide antibiotics	12	0.0033	0.0015	0.0058	22	0.0055	0.0032	0.0084	40	0.0111	0.0078	0.0149	62	0.0168	0.0128	0.0212

*N<5, **Omitted to mask not to recalculate number; IPD: individual participant data; LCL: lower confidence limit; UCL: upper confidence limit; CI: confidence interval.

Table S8: Potentially interacting prescriptions issued in the 30-day period prior to the index date by Charlson comorbidity score by combining CPRD GOLD and Aurum data using two-stage IPD meta-analysis

	Charlson index score 0				Charlson index score 1				Charlson index score 2				Charlson index score 3				Charlson index score ≥4			
	N	Proportion	95% CI		N	Proportion	95% CI		N	Proportion	95% CI		N	Proportion	95% CI		N	Proportion	95% CI	
			LCL	UCL			LCL	UCL			LCL	UCL			LCL	UCL			LCL	UCL
Drugs																				
≥1 drug	346	0.0780	0.0703	0.0861	553	0.2300	0.2133	0.2470	652	0.2804	0.2623	0.2989	600	0.3559	0.3331	0.3789	1474	0.4742	0.4567	0.4918
≥2 drugs	29	0.0064	0.0042	0.0090	48	0.0197	0.0144	0.0257	89	0.0379	0.0305	0.0461	106	0.0622	0.051	0.0743	318	0.102	0.0916	0.1130
Specific drugs																				
Statins	268	0.0604	0.0535	0.0676	438	0.1821	0.1669	0.1978	525	0.2257	0.2089	0.2429	481	0.2852	0.2638	0.307	1231	0.3960	0.3789	0.4133
Fibrates	*	**	**	**	*	**	**	**	19	0.0079	0.0045	0.012	16	0.0089	0.0047	0.0142	31	0.0096	0.0064	0.0135
Verapamil	8	0.0016	0.0005	0.0031	12	0.0047	0.0022	0.0081	7	0.0026	0.0008	0.0054	10	0.0055	0.0023	0.0099	28	0.0086	0.0056	0.0123
Diltiazem	20	0.0043	0.0025	0.0066	21	0.0085	0.0051	0.0127	48	0.0202	0.0148	0.0264	44	0.0257	0.0185	0.0339	106	0.0338	0.0277	0.0405
Digoxin	46	0.0102	0.0074	0.0134	83	0.0384	0.031	0.0465	107	0.0456	0.0374	0.0545	120	0.0752	0.0630	0.0883	323	0.1034	0.0929	0.1144
Amiodarone	*	**	**	**	9	0.0035	0.0013	0.0064	13	0.0053	0.0026	0.0088	10	0.0053	0.0022	0.0097	59	0.0186	0.0141	0.0238
Oral ketoconazole	*	**	**	**	*	**	**	**	7	0.0024	0.0006	0.0051	*	**	**	**	5	0.0012	0.0002	0.0030
Macrolide antibiotics	27	0.0059	0.0038	0.0085	21	0.0083	0.005	0.0125	21	0.0087	0.0052	0.0131	27	0.0150	0.0096	0.0216	40	0.0126	0.0089	0.0170

*N<5, **Omitted to mask not to recalculate number; IPD: individual participant data; LCL: lower confidence limit; UCL: upper confidence limit; CI: confidence interval.

Table S9: Sensitivity analysis for simvastatin and atorvastatin to estimate rates (per 10,000 person-years) and hazard ratios with 95% confidence intervals by combining CPRD GOLD and Aurum data using two-stage IPD meta-analysis

**Statin subtype**	**Diarrhoea**	**Nausea/vomiting**	**Neuropathy**	**Bone marrow suppression**	**Myalgia**	**Myocardial infarction**
**Simvastatin**						
Rate (95% CI)						
CPRD GOLD	2351.6 (1392.7, 3970.6)	664.4 (249.4, 1770.1)	166.1 (23.4, 1179.1)	332.4 (83.1, 1300)	664.4 (249.4, 1770.1)	332.9 (83.3, 1331.0)
Aurum	586.2 (386.0, 890.2)	264.7 (142.4, 491.9)	--	26.3 (3.7, 187.0)	26.4 (3.7, 187.0)	185.9 (88.6, 390.0)
Combined	*1006.1 (725.7, 1394.8)*	*344.3 (203.9, 581.3)*	*166.1 (23.4, 1179.1)*	*144.6 (46.9, 445.9)*	*348.4 (145.0, 837.0)*	*211.6 (110.1, 406.7)*
HR (95% CI)						
CPRD GOLD	1.22 (0.67, 2.23)	3.51 (0.92, 13.45)	1.05 (0.12, 9.45)	8.83 (0.71, 109.27)	3.95 (1.05, 14.90)	1.12 (0.23, 5.47)
Aurum	0.81 (0.51, 1.27)	1.09 (0.55, 2.18)	--	0.44 (0.06, 3.40)	0.46 (0.06, 3.58)	0.79 (0.35, 1.76)
Combined	*0.94 (0.66, 1.35)*	*1.39 (0.75, 2.58)*	*1.05 (0.12, 9.45)*	*1.42 (0.29, 6.78)*	*2.10 (0.69, 6.40)*	*0.85 (0.41, 1.74)*
**Atorvastatin**						
Rate (95% CI)						
CPRD GOLD	2032.6 (969.0, 4263.5)	--	--	--	575.4 (143.9, 2300.6)	286.9 (40.4, 2036.4)
Aurum	1040.7 (685.2, 1580.5)	424.3 (220.8, 815.4)	--	93.8 (23.5, 375.0)	--	477.1 (256.7, 886.7)
Combined	*1223.2 (850.0, 1760.2)*	*424.3 (220.8, 815.4)*	--	*93.8 (23.5, 375.0)*	*575.4 (143.9, 2300.6)*	*455.5 (252.3, 822.5)*
HR (95% CI)						
CPRD GOLD	1.11 (0.50, 2.45)	--	--	--	3.69 (0.71, 19.04)	1.13 (0.14, 9.16)
Aurum	1.54 (0.98, 2.41)	1.88 (0.92, 3.85)	--	1.65 (0.37, 7.40)	--	2.38 (1.19, 4.76)
Combined	*1.42 (0.96, 2.10)*	*1.88 (0.92, 3.85)*	--	*1.65 (0.37, 7.40)*	*3.69 (0.71, 19.04)*	*2.21 (1.14, 4.27)*

Each model was adjusted for age and gender for a given exposure; --: insufficient events to calculate estimates; HR: hazard ratio; CI: confidence interval; Ref.: reference category; IPD: individual participant data.

Table S10: Sensitivity analysis imputing missing BMI to estimate hazard ratios with 95% confidence intervals by combining CPRD GOLD and Aurum data using two-stage IPD meta-analysis

Covariate	Diarrhoea	Nausea and vomiting	Neuropathy	Bone marrow suppression	Myalgia	Myocardial infarction
	**HR (95% CI)**	**HR (95% CI)**	**HR (95% CI)**	**HR (95% CI)**	**HR (95% CI)**	**HR (95% CI)**
**BMI**						
Normal	1 (Ref.)	1 (Ref.)	1 (Ref.)	1 (Ref.)	1 (Ref.)	1 (Ref.)
Overweight	0.87 (0.61, 1.24)	1.01 (0.51, 2.00)	1.27 (0.28, 5.82)	0.50 (0.11, 2.23)	1.74 (0.37, 8.24)	1.18 (0.57, 2.46)
Obese	0.86 (0.60, 1.22)	0.68 (0.34, 1.36)	--	1.01 (0.26, 3.93)	2.34 (0.51, 10.65)	1.46 (0.71, 2.99)

Each model was adjusted for age and gender for a given exposure; --: insufficient events to calculate estimates; BMI categories: normal (18.5–24.9 kg/m^2), overweight (25–29.9 kg/m^2) and obese (≥30 kg/m^2); HR: hazard ratio; CI: confidence interval; Ref.: reference category; IPD: individual participant data.
